# nNOS in Erbb4-positive neurons regulates GABAergic transmission in mouse hippocampus

**DOI:** 10.1038/s41419-024-06557-1

**Published:** 2024-02-23

**Authors:** Chaofan Wan, Yucen Xia, Jinglan Yan, Weipeng Lin, Lin Yao, Meng Zhang, Inna Gaisler-Salomon, Lin Mei, Dong-Min Yin, Yongjun Chen

**Affiliations:** 1 Research Institute of Acupuncture and Moxibustion, Shandong University of Traditional Chinese Medicine, Jinan, 250355 China; 2 Department of Rehabilitation, School of Health Science, Guangdong Pharmaceutical University, Guangzhou, 510006 China; 3 South China Research Center for Acupuncture and Moxibustion, Medical College of Acu-Moxi and Rehabilitation, Guangzhou University of Chinese Medicine, Guangzhou, 510006 China; 4 Joint Center for Translational Medicine, Shanghai Fifth People’s Hospital, Fudan University and School of Life Science, East China Normal University, Shanghai, 200062 China; 5 School of Psychological Sciences, The Integrated Brain and Behavior Research Center (IBBRC), University of Haifa, Haifa, 3498838 Israel; 6 Chinese Institute for Medical Research, Beijing, 100069 China; 7 Capital Medical University, Beijing, 100069 China; 8 Chinese Institute for Brain Research, Beijing, 102206 China; 9 Guangdong Province Key Laboratory of Psychiatric Disorders, Southern Medical University, Guangzhou, 510515 China

**Keywords:** Synaptic transmission, Molecular neuroscience

## Abstract

Neuronal nitric oxide synthase (nNOS, gene name Nos1) orchestrates the synthesis of nitric oxide (NO) within neurons, pivotal for diverse neural processes encompassing synaptic transmission, plasticity, neuronal excitability, learning, memory, and neurogenesis. Despite its significance, the precise regulation of nNOS activity across distinct neuronal types remains incompletely understood. Erb-b2 receptor tyrosine kinase 4 (ErbB4), selectively expressed in GABAergic interneurons and activated by its ligand neuregulin 1 (NRG1), modulates GABA release in the brain. Our investigation reveals the presence of nNOS in a subset of GABAergic interneurons expressing ErbB4. Notably, NRG1 activates nNOS via ErbB4 and its downstream phosphatidylinositol 3-kinase (PI3K), critical for NRG1-induced GABA release. Genetic removal of nNos from Erbb4-positive neurons impairs GABAergic transmission, partially rescued by the NO donor sodium nitroprusside (SNP). Intriguingly, the genetic deletion of nNos from Erbb4-positive neurons induces schizophrenia-relevant behavioral deficits, including hyperactivity, impaired sensorimotor gating, and deficient working memory and social interaction. These deficits are ameliorated by the atypical antipsychotic clozapine. This study underscores the role and regulation of nNOS within a specific subset of GABAergic interneurons, offering insights into the pathophysiological mechanisms of schizophrenia, given the association of Nrg1, Erbb4, Pi3k, and Nos1 genes with this mental disorder.

## Introduction

Neuronal nitric oxide synthase (nNos), also referred to as Nos1, stands as the principal enzyme responsible for generating 90% of neuronal nitric oxide (NO) in the central nervous system. The expression and activation of nNos are intricately regulated by key signaling proteins, including cyclic adenosine monophosphate (cAMP) response element-binding protein (CREB), calmodulin (CaM), heat shock protein 90 (HSP90)/HSP70, and AKT [1]. A wealth of evidence underscores the pivotal role of nNOS/NO in modulating various physiological functions such as synaptic transmission, neuronal plasticity, excitability, recognition, learning, memory, and neurogenesis [1–3]. Recent revelations have linked nNOS to psychiatric disorders, including depression and anxiety [4–6]. Disruption of nNOS-CAPON coupling contributes to an anxiolytic effect [7] and prevents addiction relapse [8] in rodents, while the serotonin transporter (SERT)-nNOS complex is associated with depression-like behaviors [6]. However, the specific function and regulation of nNOS in a cell type-specific manner remain incompletely understood.

Remarkably, nNos predominantly finds expression in a subset of GABAergic interneurons in both juvenile and mature hippocampus, as well as the neocortex [9, 10]. A substantial body of evidence supports the involvement of nNOS/NO signaling in synaptic transmission. Notably, glutamate and γ-aminobutyric acid (GABA) release stimulated by N-methyl-D-aspartate in the brain are significantly reduced in Nos knockout mice in vivo [11]. Earlier studies indicate that NO increases the GABAergic spontaneous postsynaptic current frequency from amacrine cells in the inner retina [12, 13]. Furthermore, NO potentiates GABA release in the nucleus tractus solitarii [14] and the hypothalamic paraventricular nucleus [15]. These findings suggest a potential role of nNOS/NO in modulating GABAergic transmission.

ErbB4, a receptor tyrosine kinase, incorporates an epidermal growth factor (EGF) domain, and its activation relies on neuregulin 1 (NRG1), a ligand binding to ErbB4. The majority of Erbb4-positive (Erbb4^+^) cells in cortex, hippocampus and amygdala are GABAergic [16]. Previous investigations have revealed that NRG1-ErbB4 signaling dynamically modulates GABA release, influencing pyramidal neuron activity [17], synaptic plasticity [18], the excitation/inhibition (E/I) balance [19, 20] and hippocampal-prefrontal synchrony [21]. Additionally, NRG1 may regulate GABA release from synaptosomes in the absence of their neural circuit [22]. Importantly, NRG1 has been shown to enhance NO production in adult rat ventricular myocytes and rostral ventrolateral medulla [23, 24]. Interestingly, ErbB4 and nNOS are co-expressed in some GABAergic interneurons in mouse hippocampus [25]. These studies propose that nNOS might be a downstream target of ErbB4 signaling to regulate GABAergic transmission. Nevertheless, the specific functions of nNOS in Erbb4-positive GABAergic interneurons have not been thoroughly explored.

Here we provide evidence demonstrating that the majority of Erbb4-positive neurons in mouse hippocampus express nNOS. Activation of nNOS via NRG1-ErbB4 signaling promotes GABA release. Genetic deletion of nNos from Erbb4-positive neurons (Erbb4-nNos^−/−^) impedes GABAergic transmission, resulting in an elevated E/I ratio and heightened pyramidal neuron activity. Furthermore, Erbb4-nNos^−/−^ mice manifest several schizophrenia-relevant behavioral deficits, including hyperactivity, impaired sensorimotor gating, and deficient working memory and social interaction. These deficits can be mitigated by clozapine administration. In summary, our findings provide compelling evidence for a cell-type-specific function and regulation of nNOS in the mouse hippocampus.

## Results

### nNOS expression in Erbb4-positive neurons of mouse hippocampus

**Fig. 1:**
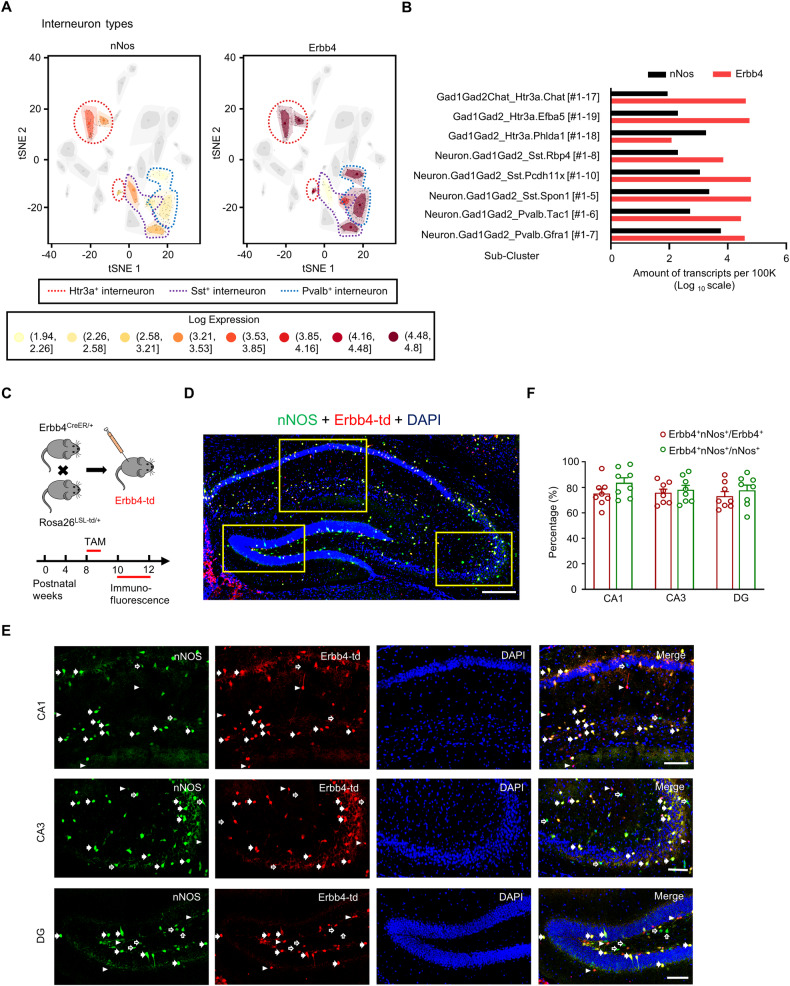
nNOS expression in Erbb4-positive neurons of mouse hippocampus. **A** tSNE plot showing the clustering of interneuron types that express nNos and Erbb4 in hippocampus analyzed by Single cell RNA-seq from mice. **B** Quantification of nNos and Erbb4 expression in different sub-clusters of interneurons. **C** Experimental design for (**D**-**F**). Top: Breeding diagram for the generation of Erbb4^CreER/+^; Rosa26^LSL-td/+^mice (Erbb4-td mice). Bottom: Time point of Tamoxifen injection and Immunofluorescence. **D** Images showing Erbb4^+^ neurons (red) in the hippocampus region co-labeled with nNos (green) from Erbb4-td mice. Scale bar = 250 μm. **E** Representative micrographs of nNos^+^ neurons (green) in CA1, CA3 and DG. Solid arrows marked the neurons co-expressed Erbb4 and nNos, hollow arrows marked neurons express nNos only and arrowheads marked Erbb4^+^ positive neurons. Scale bar = 100 μm. **F** Quantification of the radio of Erbb4^+^ nNos^+^/Erbb4^+^ neurons and Erbb4^+^ nNos^+^/nNos^+^ neurons in hippocampal CA1, CA3 and DG. *n* = 5 mice. Data are mean ± SEM.

Utilizing single-cell RNA-sequencing data obtained from the mouse hippocampus [26], we identified co-expression of nNos with Erbb4 in specific subpopulations of GABAergic neurons, including those positive for serotonin receptor 3 A (Htr3a), somatostatin (Sst), and parvalbumin (Pvalb) (Fig. 1A and B). The exact proportion of Erbb4-positive neurons that co-express nNos, and vice versa, remains uncertain. To address this, we performed immunostaining with anti-nNos antibodies in the hippocampus of Erbb4-reporter mice expressing tdTomato (abbreviated to Erbb4-td thereafter) in Erbb4-positve cells (Fig. 1C-E) [16]. Our results revealed that 75.18 ± 3.6% of Erbb4-positive neurons co-express nNos and 83.65 ± 3.91% of the nNos-positive neurons co-express Erbb4 in the hippocampal CA1 region (Fig. 1F). Similar co-expression levels of Erbb4 and nNos were observed in CA3 and dentate gyrus (Fig. 1F). These findings collectively establish that a significant proportion of Erbb4-positive neurons within the mouse hippocampus also express nNos.

### Activation of AKT-nNOS signaling by NRG1-ErbB4 increases GABA release

**Fig. 2:**
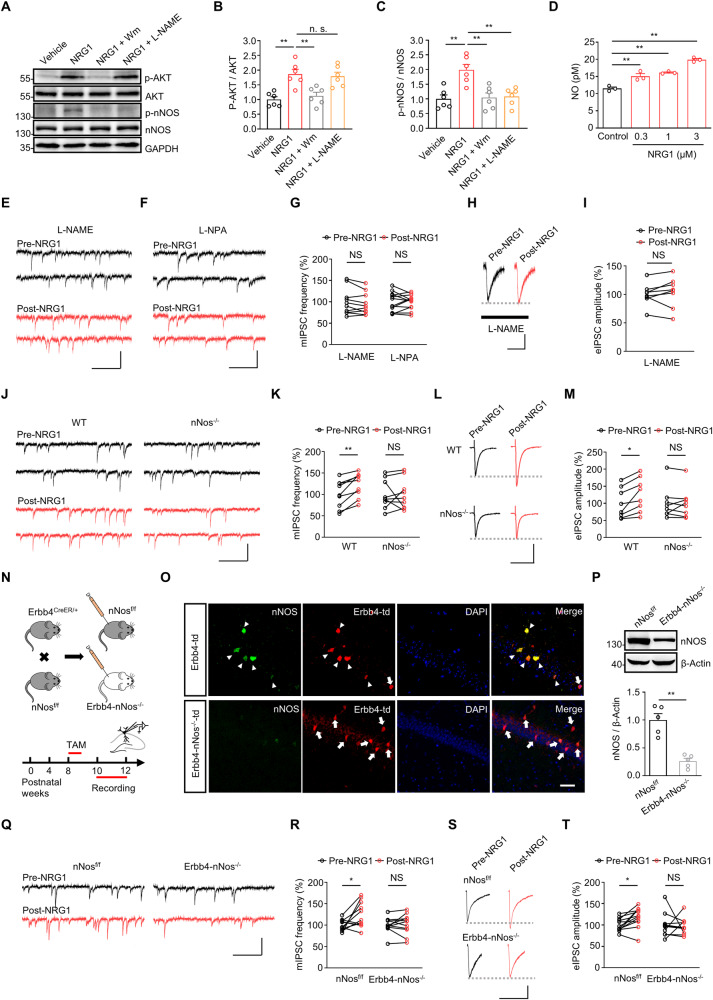
Activation of AKT-nNOS signaling by NRG1 increases GABA release. **A**-**C** Representative western blot of hippocampal tissue from different treatment **(A)** and quantification of total phospho (S473)- and total AKT (pAKT and AKT) **(B)** and phospho (1417)- and total nNOS (p-nNOS and nNOS) **(C)** protein levels in hippocampus after various treatments. **B** one-way ANOVA with Bonferroni’s multiple comparisons test, *F* (3, 20) = 11.34, *P* = 0.0001; Vehicle vs. NRG1, *P* = 0.0010; NRG1 vs. NRG1+Wm, *P* = 0.0046; NRG1 vs. NRG1 + L-NAME, *P* > 0.9999. **C** one-way ANOVA with Bonferroni’s multiple comparisons test, *F* (3, 20) = 9.753, *P* = 0.0004; Vehicle vs. NRG1, *P* = 0.0010; NRG1 vs. NRG1+Wm, *P* = 0.0017; NRG1 vs. NRG1 + L-NAME, *P* = 0.0025. n = 6 mice for each group. **D** Dose-dependent potentiation of NRG1 on NO production in cultured hippocampal neurons. One-way ANOVA with Bonferroni’s multiple comparisons test, *F* (3, 8) = 51.64, *P* < 0.0001; Control vs. 0.3 μM, *P* = 0.0024; Control vs. 1 μM, *P* = 0.0004; Control vs. 3 μM, *P* < 0.0001. n = 3 samples for each group. **E**, **F** Representative traces of mIPSCs in CA1 pyramidal neurons before and after NRG1 treatment in the presence of 300 μM L-NAME **E** or 200 nM L-NPA **(F)**. Scale bar = 250 ms, 50 pA. **G** Quantification of mean values of mIPSC frequency. L-NAME, paired *t* test, *t* = 1.198, *P* = 0.2615. n = 10 neurons from 4 mice; L-NPA, paired *t* test, *t* = 0.4065, *P* = 0.6922. n = 12 neurons from 3 mice. **H**, **I** Representative traces of eIPSCs **(H)** and quantification of mean values of eIPSC amplitude **(I)** before and after 5 nM NRG1 treatment in the presence of L-NAME in artificial cerebrospinal fluid (ACSF). Scale bars = 100 ms, 200 pA. Paired *t* test, *t* = 0.5909, *P* = 0.5732. n = 8 neurons from 3 mice. **J**, **K** Representative traces of mIPSCs **(J)** and quantification of mean values of mIPSC frequency **(K)** from WT (Wildtype) and nNos^−/−^ mice before and after 5 nM NRG1 treatment. Scale bar = 250 ms, 50 pA. WT: paired *t* test, *t* = 3.894, *P* = 0.0046; nNos^−/−^: paired *t* test, *t* = 0.3907, *P* ^=^ 0.7062. n = 9 neurons from 3 mice for each group. **L**, **M** Representative traces **(L)** of eIPSCs from WT and nNos^−/−^ mice before and after 5 nM NRG1 treatment, and quantification of mean values of eIPSC amplitude **(M)**. Scale bars = 100 ms, 200 pA. WT: paired *t* test, *t* = 2.984, *P* = 0.0204; nNos^−/−^: paired *t* test, *t* = 0.2844, *P* ^=^ 0.7844. *n* = 8 neurons from 3 mice for each group. **N** Scheme of experimental design. Top: Breeding diagram for the generation of Erbb4^CreER/+^; nNos^f/f^ mice (Erbb4-nNos^−/−^ mice). Bottom: Time frame of Tamoxifen injection and electrophysiological recording. **O** Fluorescence images for nNos immunoreactivity (green) in the hippocampal CA1 region of Erbb4-nNos^−/−^-td and Control (Erbb4-td) mice. Scale bar = 50 μm. **P** Representative western blot (top) and quantification of nNOS protein levels (bottom) in hippocampus. Unpaired *t* test, *t* = 5.865, *P* = 0.0004. *n* = 5 mice per group. **Q** Representative traces of mIPSCs from nNos^f/f^ and Erbb4-nNos^−/−^ mice before and after 5 nM NRG1 treatment. Scale bar = 250 ms, 50 pA. **R** Quantification of mean values of mIPSC frequency before and after 5 nM NRG1 treatment. nNos^f/f^: paired *t* test, *t* = 2.36, *P* = 0.04; Erbb4-nNos^−/−^: paired *t* test, *t* = 0.4573, *P* ^=^ 0.6572. *n* = 11 neurons from 5 nNos^f/f^ mice; *n* = 11 neurons from 6 Erbb4-nNos^−/−^ mice. **S** Representative traces of eIPSCs from nNos^f/f^ and Erbb4-nNos^−/−^ mice before and after 5 nM NRG1 treatment. Scale bars = 50 ms, 100 pA. **T** Quantification of mean values of eIPSC amplitude. nNos^f/f^: paired *t* test, *t* = 3.035, *P* = 0.0114; Erbb4-nNos^−/−^: paired *t* test, *t* = 0.3316, *P* ^=^ 0.7478. *n* = 12 neurons from 5 nNos^f/f^ mice. *n* = 10 neurons from 6 Erbb4-nNos^−/−^ mice. Data are mean ± SEM. NS, not significant, *P < 0.05, **P < 0.01.

NRG1-ErbB4 signaling induces the activation of several serine-threonine protein kinases, including phosphatidylinositol 3-kinase (PI3K)-protein kinase B (AKT), c-Jun N-terminal kinase (JNK), and extracellular-regulated kinase (ERK) [27, 28]. As seen in Fig. 2A-C and Fig. S6, NRG1 increased the levels of phopho-Ser473-AKT (an active form of AKT) without affecting the total protein levels of AKT in hippocampal slices, a response impeded by Wortmannin. This observation confirms that NRG1 activates PI3K-AKT signaling in hippocampal slices. AKT has been shown to phosphorylate nNOS at Serine 1417 to increase nitric oxide (NO) production and intracellular Ca^2+^ levels in mouse intestine [29]. To explore whether NRG1 can activate nNOS through AKT, NRG1 was perfused in hippocampal slices for 5 minutes, and the active form of nNOS was assessed using anti-phospho-Ser^1417^-nNOS antibody [30]. As depicted in Fig. 2A-C and Fig. S6, NRG1 upregulated phospho-Ser^1417^-nNOS levels, without altering total nNOS protein levels, and this effect was prevented by the NOS inhibitor Nω-Nitro-L-arginine methyl ester (L-NAME). These results indicate that acute NRG1 treatment activates nNOS in hippocampal slices. Correspondingly, NRG1 induced a dose-dependent increase in NO production from mouse hippocampal neurons (Fig. 2D). Notably, the elevation of phospho-Ser^1417^-nNOS after NRG1 treatment was abolished by Wortmannin (Fig. 2A-C, Fig. S6). Together, our findings provide compelling evidence that NRG1 can activate nNOS through PI3K-AKT in hippocampal slices.

Prior investigations have shown that acute NRG1 treatment enhances GABAergic transmission in CA1 pyramidal neurons, an effect obstructed by ecto-ErbB4 or the ErbB4 inhibitor AG1478 [17, 18, 22]. To validate the impact of NRG1 on GABAergic transmission, we recorded miniature inhibitory postsynaptic currents (mIPSCs) and evoked inhibitory postsynaptic currents (eIPSCs) of CA1 pyramidal neurons in mouse hippocampus before and 5 minutes after treatment with different concentrations of NRG1 ECD peptide (abbreviated to NRG1 thereafter). To assess whether nNOS activation is crucial for enhanced GABA release with NRG1 treatment, hippocampal slices were perfused with L-NAME or Nω-propyl-L-Arginine (L-NPA, an nNOS inhibitor) before NRG1 incubation. Intriguingly, both L-NAME and L-NPA abolished the incremental effects of NRG1 on mIPSC frequency and eIPSC amplitude (Fig. 2E-I), rather than mIPSC amplitude (Fig. S1A). Consistent with our previous studies [18, 22], NRG1 diminished the paired-pulse ratio (PPR) of eIPSC, suggesting NRG1 promotes GABA release via a presynaptic mechanism (Fig. S2A-B). Concurrently, the application of L-NPA blocked the effect of NRG1 on PPR of eIPSC (Fig. S2C-D). These results affirm that nNOS activation is in dispensable for NRG1 to enhance GABA release. To reinforce this conclusion, we examined GABA release in nNos null mutant (nNos^−/−^) mice [31]. Acute treatment with NRG1 augmented the mIPSC frequency and eIPSC amplitude of CA1 pyramidal neurons in wildtype (WT) littermates, but not nNos^−/−^ mice (Fig. 2J-M and S1B). It is noteworthy that NO inhibits GABA transaminase (GABAT), preventing GABA degradation [32], leading to subsequent increases in extracellular GABA concentration affecting mIPSC frequency and amplitude [33].

To rule out the potential influence of GABAT on enhanced mIPSC frequency induced by NRG1, we measured mIPSC before and after NRG1 treatment in the presence of 40 µM Vigabatrin, a GABAT inhibitor. As shown in Fig. S3, pretreatment with Vigabatrin failed to impede the increased mIPSC frequency caused by NRG1. Together these results indicate that NRG1 enhances GABAergic transmission through increasing GABA release rather than impairing GABA degradation.

To further investigate whether activation of nNos in Erbb4-positive neurons is important for modulating GABA release, we generated mutant mice in which nNos was selectively deleted from Erbb4-positive neurons (abbreviated to Erbb4-nNos^−/−^ mice) using the Cre-LoxP strategy (Fig. 2N). The activity of Cre recombinase in Erbb4^Cre-ER/+^ mice, used in this study, is tamoxifen dependent [34]. To eliminate the potential effects of tamoxifen, both control (nNos^f/f^) and Erbb4-nNos^−/−^ mice underwent tamoxifen treatment. Immunostaining and western blots confirmed the downregulation of nNOS protein in Erbb4-positive cells and hippocampal tissue from Erbb4-nNos^−/−^ mice, compared with controls (Fig. 2O-P and Fig. S6). Acute treatment with NRG1 increased the mIPSC frequency and eIPSC amplitude of CA1 pyramidal neurons in control mice (Fig. 2Q-T and S1C), consistent with NRG1 effects in WT mice (Fig. 2J-M and S1B). However, NRG1 did not increase mIPSC frequency or eIPSC amplitude of CA1 pyramidal neurons in Erbb4-nNos^−/−^ mice (Fig. 2Q-T). These data demonstrate that activation of nNos from Erbb4-positive neurons is crucial for promoting GABA release.

### Genetic deletion of nNos from Erbb4-positive neurons impairs GABAergic transmission and increases the E/I ratio

**Fig. 3:**
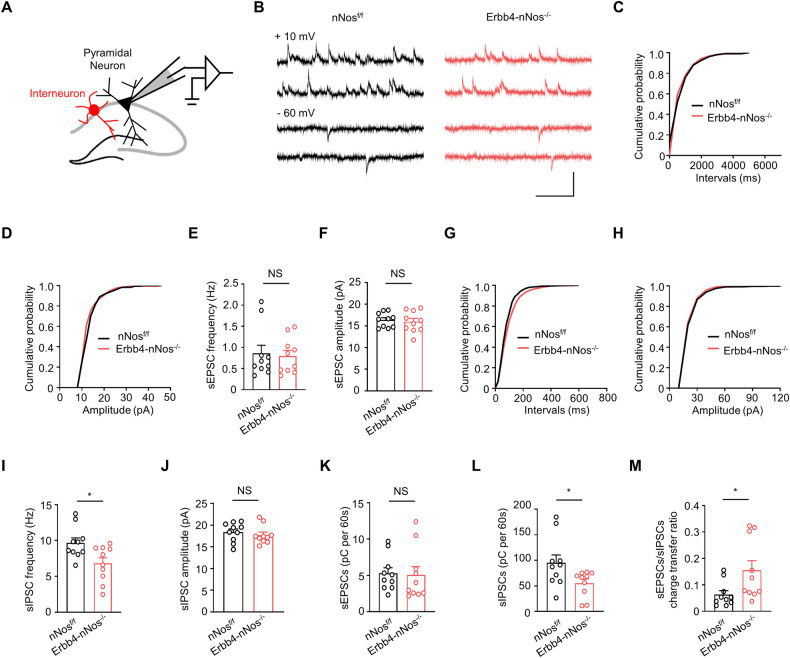
Genetic deletion of nNos from Erbb4-positive neurons impairs GABAergic transmission and increases the E/I ratio. **A** Schematic representation of whole-cell recordings from pyramidal neurons in hippocampus. **B** Representative traces of sEPSCs (- 60 mV) and sIPSCs (+ 10 mV) in CA1 pyramidal neurons from nNos^f/f^ and Erbb4-nNos^−/−^ mice. Scale bar = 250 ms, 50 pA. **C**, **D** Cumulative plots of sEPSC interevent intervals **(C)** and amplitude **(D)**. **E**, **F** Quantification of mean values of sEPSC frequency **(E)** and amplitude **(F)**. **E** Unpaired t test, *t* = 0.2703, *P* = 0.7900. **F** Unpaired t test, *t* = 0.4205, *P* = 0.6791. **G**, **H** Cumulative plots of sIPSC interevent intervals **(G)** and amplitude **(H)**. **I**, **J** Quantification of mean values of sIPSC frequency **(I)** and amplitude **(J)**. **I** Unpaired t test, *t* = 2.710, *P* = 0.0143. **J** Unpaired t test, *t* = 0.7217, *P* = 0.4798. **K** Quantification of sEPSCs from nNos^f/f^ and Erbb4-nNos^−/−^ mice. Unpaired t test, *t* = 0.1473, *P* = 0.8845. **L** Quantification of sIPSCs from nNos^f/f^ and Erbb4-nNos^−/−^ mice. Unpaired t test, *t* = 2.273, *P* = 0.0355. **M** Quantification of sEPSC/sIPSC charge transfer ratios from nNos^f/f^ and Erbb4-nNos^−/−^ mice. Unpaired t test, *t* = 2.351, *P* = 0.0303. **C**-**M** *n* = 10 neurons from 3 nNos^f/f^ mice. *n* = 10 neurons from 3 Erbb4-nNos^−/−^ mice. Data are mean ± SEM. NS, not significant, *P < 0.05, **P < 0.01.

**Fig. 4:**
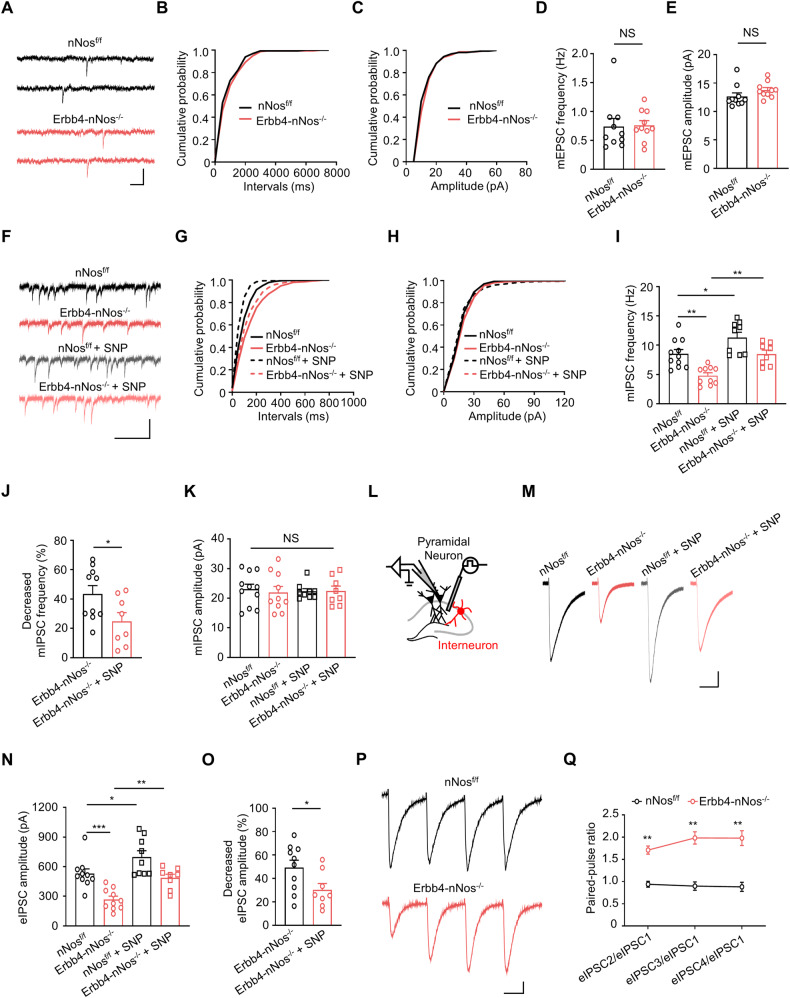
Genetic deletion of nNos from Erbb4-positive neurons impairs GABAergic transmission via a presynaptic mechanism, with no effect on glutamatergic transmission. **A** Representative traces of mEPSCs in CA1 pyramidal neurons from nNos^f/f^ and Erbb4-nNos^−/−^ mice. Scale bar = 200 ms, 20 pA. **B**, **C** Cumulative plots of mEPSC interevent intervals **(B)** and amplitude **(C)**. **D**, **E** Quantification of mean values of mEPSC frequency **(D)** and amplitude **(E)**. **D** Unpaired t test, *t* = 0.1263, *P* = 0.9009. **E** Unpaired t test, *t* = 1.499, *P* = 0.1512. n = 10 neurons from 3 mice for each group. **F** Representative traces of mIPSCs in CA1 pyramidal neurons from nNos^f/f^ and Erbb4-nNos^−/−^ mice with or without the NO donor Sodium Nitroprusside (SNP, 100 μM) in ACSF. Scale bar = 250 ms, 50 pA. **G**, **H** Cumulative plots of mIPSC interevent intervals **(G)** and amplitude **(H)**. **I**-**K** Quantification of mean values of mIPSC frequency **(I)**, decreased mIPSC frequency **(J)** and amplitude **(K)**. **I** One-way ANOVA with Bonferroni’s multiple comparisons test, *F* (3, 34) = 13.02, *P* < 0.0001; nNos^f/f^ vs. Erbb4-nNos^−/−^, *P* = 0.0021; nNos^f/f^ vs. Erbb4-nNos^−/−^ + SNP, *P* = 0.0306; Erbb4-nNos^−/−^ vs. Erbb4-nNos^−/−^ + SNP, *P* = 0.0156. **J** Unpaired t test, *t* = 2.273, *P* = 0.0372. **K** one-way ANOVA with Bonferroni’s multiple comparisons test, *F* (3, 34) = 0.0798, *P* = 0.9705. *n* = 11 neurons from 5 nNos^f/f^ mice. *n* = 10 neurons from 5 Erbb4-nNos^−/−^ mice. n ^=^ 9 neurons from 3 nNos^f/f^ mice when applicating with SNP. *n* = 8 neurons from 3 Erbb4-nNos^−/−^ mice when applicating with SNP. **L** Schematic representation of the position of recording and stimulus pipette. **M** Representative traces of eIPSCs in CA1 pyramidal neurons from nNos^f/f^ and Erbb4-nNos^−/−^ mice with or without 100 μM SNP in ACSF. Scale bars = 25 ms, 100 pA. **N**, **O** Quantification of mean values of eIPSC amplitude (**N**) and decreased eIPSC amplitude (**O**). **N** One-way ANOVA with Bonferroni’s multiple comparisons test, *F* (3, 33) = 14.32, *P* < 0.0001; nNos^f/f^ vs. Erbb4-nNos^−/−^, *P* = 0.0009; nNos^f/f^ vs. Erbb4-nNos^−/−^ + SNP, *P* = 0.0484; Erbb4-nNos^−/−^ vs. Erbb4-nNos^−/− +^ SNP, *P* = 0.0128. **O** Unpaired t test, *t* = 2.225, *P* = 0.0408. *n* = 10 neurons from 3 nNos^f/f^ mice. *n* = 10 neurons from 3 Erbb4-nNos^−/−^ mice. *n* = 9 neurons from 3 nNos^f/f^ mice when applicating with SNP. *n* = 8 neurons from 3 Erbb4-nNos^−/−^ mice when applicating with SNP. **P**, **Q** Representative traces (**P**) of PPR of eIPSCs from nNos^f/f^ and Erbb4-nNos^−/−^ mice, quantification of mean values of PPR (**Q**). Scale bar = 25 ms, 100 pA. **Q** Two-way Repeated Measures ANOVA with Bonferroni’s multiple comparisons test, Genotype *F* (1, 18) = 49.91, *P* < 0.0001; eIPSC2/eI*P*SC1: *P* < 0.0001; eIPSC3/eIPSC1: *P* < 0.0001; eIPSC4/eIPSC1: *P* < 0.0001. *n* = 10 neurons from 3 mice for each group. Data are mean ± SEM. NS, not significant, *P < 0.05, **P < 0.01.

Both ErbB4 and nNos are predominantly expressed in GABAergic interneurons of mouse hippocampus [10, 16]. To elucidate the functional consequences of nNOS mutation in Erbb4-nNos^−/−^ mice, we performed whole-cell patch-clamp recordings from hippocampal CA1 pyramidal neurons (Fig. 3A). Initially, we recorded spontaneous excitatory postsynaptic currents (sEPSC) and spontaneous inhibitory postsynaptic currents (sIPSC) to verify whether the spontaneous synaptic transmission was altered. nNos deletion had no effect on the frequency and the amplitude of sEPSC (Fig. 3B-F) but decreased the frequency, rather than the amplitude, of sIPSC (Fig. 3B, G-J), resulting in an increased E/I ratio (Fig. 3K-M). To study whether basal neurotransmission was affected by this deletion, we examined miniature excitatory postsynaptic currents (mEPSC) and miniature inhibitory postsynaptic currents (mIPSC) in pyramidal neurons. Both the frequency and amplitude of mEPSC in pyramidal neurons were similar between Erbb4-nNos^−/−^ mice and nNos^f/f^ mice (Fig. 4A-E). However, the frequency, but not the amplitude, of mIPSC in pyramidal neurons decreased in Erbb4-nNos^−/−^ mice compared with control mice (Fig. 4F-K). Intriguingly, the application of the NO donor Sodium Nitroprusside (SNP) in ACSF rescued the deficits of mIPSC frequency in Erbb4-nNos^−/−^ mice (Fig. 4I and J). These results indicate that downregulation of nNos impairs GABAergic transmission and induces E/I imbalance in the hippocampus, which can be partly reversed by NO.

To investigate whether nNos deletion decreases GABAergic transmission via a presynaptic mechanism, we recorded evoked IPSCs (eIPSC) in CA1 pyramidal neurons (Fig. 4L). As expected, the amplitude of eIPSC was lower in Erbb4-nNos^−/−^ mice, compared with control mice (Fig. 4M and N). Furthermore, the paired-pulse ratio (PPR) of eIPSC increased in Erbb4-nNos^−/−^ mice (Fig. 4P and Q), indicating a decreased presynaptic GABA release probability. Similar results were also observed when recording eIPSC from Erbb4-nNos^−/−^ mouse slices. The application of SNP increased the amplitude of eIPSC (Fig. 4N and O), indicating that NO can ameliorate the impaired presynaptic GABA release in Erbb4-nNos^−/−^ mice. Taken together, these results demonstrated that nNOS in Erbb4-positive cells is essential for GABAergic transmission via a presynaptic mechanism.

### Genetic deletion of nNos from Erbb4-positive neurons induces hyperactivity of pyramidal neurons

To rule out the potential effect of nNos deletion on Erbb4-positive neuron intrinsic excitability, we performed whole-cell current clamp recordings from Erbb4-positive neurons in CA1 (Fig. S4A and B, S5A and B). Erbb4-positive neurons were categorized into two types, namely, fast-spiking neurons and regular-spiking neurons, based on their firing patterns (Fig. S4C and S5C). Analysis of various electrophysiological parameters, including action potential (AP) frequency, resting membrane potential, AP threshold, AP amplitude, fast afterhyperpolarization (fAHP) amplitude, input resistance, and time constant (τ), revealed no significant differences between Erbb4-nNos^−/−^ and control mice (Fig. S4D-K and S5D-K). These findings suggest that genetic deletion of nNos from Erbb4-positive neurons does not affect the excitability of Erbb4-positive neurons.

**Fig. 5:**
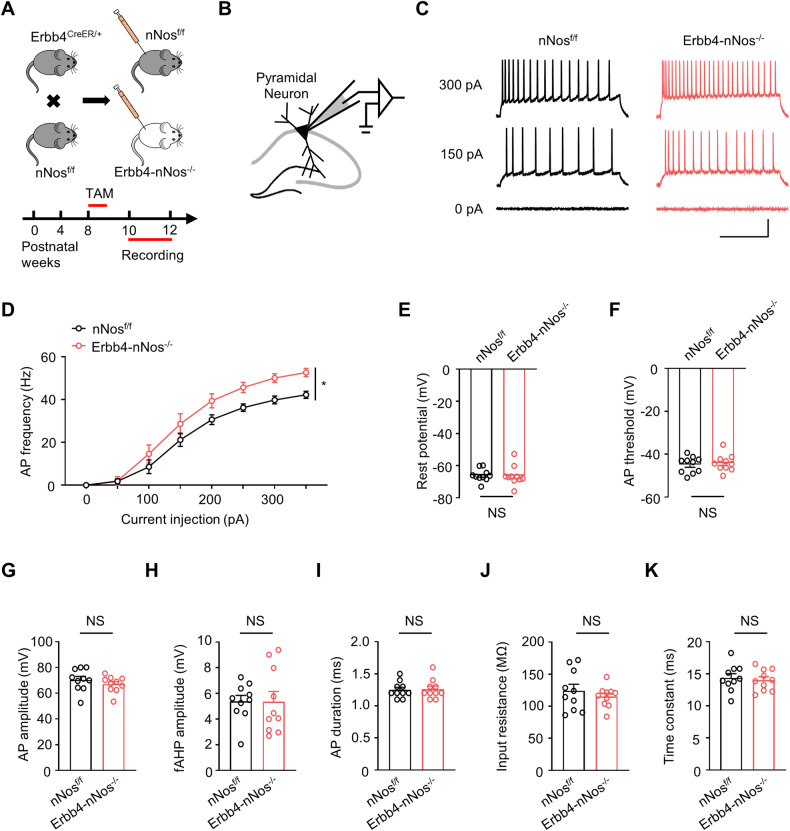
Genetic deletion of nNos from Erbb4-positive neurons increases firing rate of pyramidal neuron. **A** Scheme of experimental design. Top: Breeding diagram for the generation of Erbb4^CreER/+^; nNos^f/f^ mice (Erbb4-nNos^−/−^ mice). Bottom: Time frame of Tamoxifen injection and electrophysiological recording. **B** Schematic of whole-cell recordings from pyramidal neurons in hippocampus. **C** Representative traces of APs in CA1 pyramidal neurons from nNos^f/f^ and Erbb4-nNos^−/−^ mice. Scale bar = 200 ms, 30 mV. **D** Quantification of AP frequency induced by different intensity of current injection from nNos^f/f^ and Erbb4-nNos^−/−^ mice. Two-way Repeated Measures ANOVA with Bonferroni’s multiple comparisons test, Genotype *F* (1, 18) = 5.885, *P* = 0.0260. **E** Quantification of rest potential. Unpaired t test, *t* = 0.1039, *P* = 0.9184. **F** Quantification of AP threshold. Unpaired t test, *t* = 0.4382, *P* = 0.6665. **G** Quantification of AP amplitude. Unpaired t test, *t* = 0.9590, *P* = 0.3502. **H** Quantification of fAHP amplitude. Unpaired t test, *t* = 0.03651, *P* = 0.9713. **I** Quantification of AP duration. Unpaired t test, *t* = 0.1606, *P* = 0.8742. **J** Quantification of input resistance. Unpaired t test, *t* = 0.7946, *P* = 0.4372. **K** Quantification of time constant. Unpaired t test, *t* = 0.4053, *P* = 0.6900. **(D**-**K)** n = 10 neurons from 3 mice for each group. Data are mean ± SEM. NS not significant, *P < 0.05, **P < 0.01.

Deficits in GABAergic transmission may lead to disinhibition, resulting in increased excitability of local pyramidal neurons [35]. To assess the impact on pyramidal neurons, we examined AP firing rate and conducted electrophysiological recordings following the protocol used for Erbb4-positive neurons (Fig. 5A-C). In Erbb4-nNos^−/−^ mice, pyramidal neurons exhibited an upward shift in the input–output (I/O) curves of AP compared to control mice, indicating enhanced excitability. However, no other changes were observed (Fig. 5D, E-K). In summary, the downregulation of nNos increased the cell-intrinsic excitability of pyramidal neurons but had no effect on Erbb4-positive neurons.

### Genetic deletion of nNos from Erbb4-positive neurons in the hippocampus induces schizophrenia-relevant behavioral deficits

**Fig. 6:**
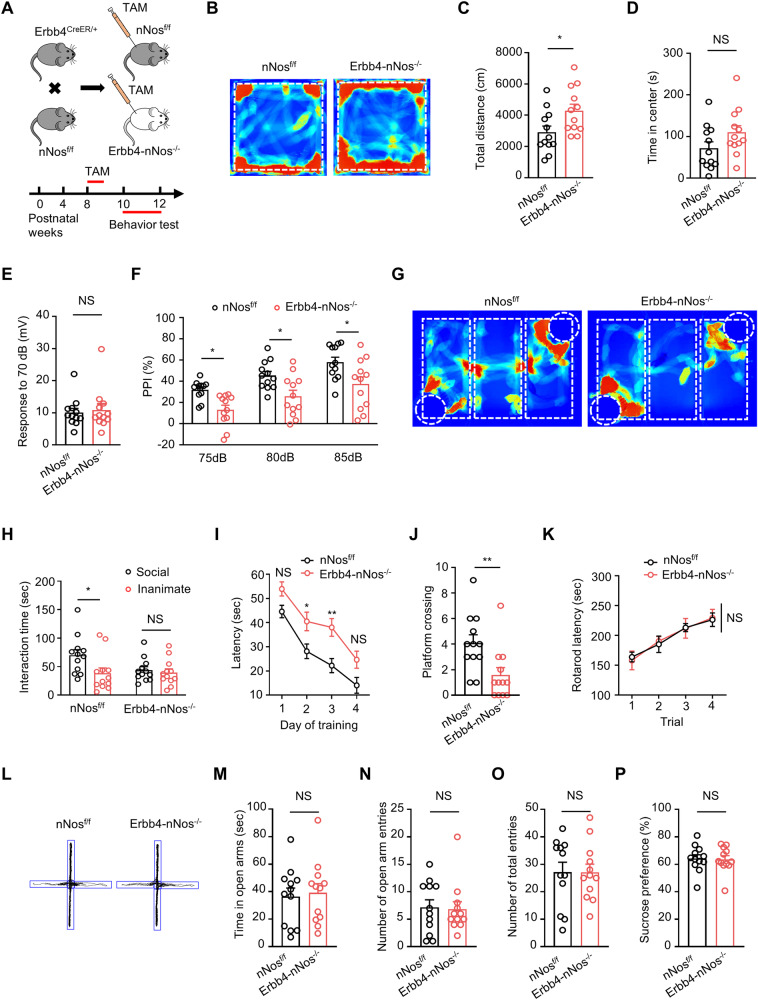
Genetic deletion of nNos from Erbb4-positive neurons in hippocampus induces schizophrenia-relevant behavioral deficits. **A** Scheme of experimental design. Top: Breeding diagram for the generation of Erbb4^CreER/+^; nNos^f/f^ mice (Erbb4-nNos^−/−^ mice). Bottom: Time frame of Tamoxifen injection and behavior testing. **B**–**D** Representative activity tracking **(B)**, total distance **(C)** and time in center **(D)** from nNos^f/f^ and Erbb4-nNos^−/−^ mice in open field test. **C** Unpaired t test, *t* = 2.383, *P* = 0.0262. **D** Unpaired t test, *t* = 1.696, *P* = 0.1040. **E**, **F** Quantification of response to 70 dB **(E)** and percentage of PPI **(F)** in prepulse inhibition test. **E** unpaired t test, *t* = 0.3513, *P* = 0.7287. **F** Two-way Repeated Measures ANOVA with Bonferroni’s multiple comparisons test, Genotype *F* (1, 22) = 10.64, *P* = 0.0036. 75 dB: *P* = 0.0204; 80 dB: *P* = 0.0159; 85 dB: *P* = 0.0100. **G**, **H** Representative activity tracking **(G)** and interaction time **(H)** in the three-chamber social test. **H** Two-way ANOVA with Bonferroni’s multiple comparisons test. nNos^f/f^ mice: *P* = 0.0163; Erbb4-nNos^−/−^ mice: *P* > 0.9999. **I**, **J** Quantification of latency (**I**) and platform crossing (**J**) in the water maze. **I** Two-way Repeated Measures ANOVA with Bonferroni’s multiple comparisons test, Genotype *F* (1, 22) = 28.10, *P* < 0.001. Day 1: *P* = 0.1772; Day 2: *P* = 0.0343; Day 3: *P* = 0.0035; Day 4: *P* = 0.0915. **J** Unpaired t test, *t* = 2.875, *P* = 0.0088. **K** Quantification of latency to fall in rotarod test. Repeated two-way ANOVA with Bonferroni’s multiple comparisons test, Genotype *F* (1, 22) = 0.001137, *P* = 0.9734. Trial 1, Trial 2, Trial 3, Trial 4: *P* > 0.9999. **L**-**O** Representative activity tracking (**L**), time in open arms (M), number of open arm entries (**N**) and number of total entries (**O**) in elevated plus maze test. **M** Unpaired t test, *t* = 0.3073, *P* = 0.7615. **N** Unpaired t test, *t* = 0.1742, *P* = 0.8633. **O** Unpaired t test, *t* = 0.01789, *P* = 0.9859. **P** Quan*t*ification of sucrose preference. Unpaired t test, *t* = 0. 1739, *P* = 0.8635. (B-P) *n* = 12 mice for each group. Data are mean ± SEM. NS, not significant, *P < 0.05, **P < 0.01.

Given that both Erbb4 and nNos mutant mice exhibit behavioral phenotypes resembling schizophrenia-like, depression-like, and anxiety-like symptoms [20, 36–38], we investigated whether the deletion of nNos from Erbb4-positive neurons resulted in behavioral deficits (Fig. 6A). A series of behavioral tests was conducted to elucidate the role of nNos in Erbb4-positive neurons. In the open-field test, Erbb4-nNos^−/−^ mice displayed an increased total distance traveled compared to controls, suggesting a schizophrenia-like locomotor hyperactivity phenotype [39] (Fig. 6B-D). However, there was no difference in the time spent in the center between genotypes. The prepulse inhibition (PPI) test, a measure of sensorimotor gating often impaired in schizophrenia patients [40], revealed a lower level of PPI in Erbb4-nNos^−/−^ mice compared to controls, indicating impaired sensorimotor gating (Fig. 6E and F). To assess sociability, a three-chamber social interaction test was performed, revealing that Erbb4-nNos^−/−^ mice spent less time engaging in social interaction with a stimulus mouse compared to control mice, indicative of impaired social activity (Fig. 6G and H). In the water maze test, Erbb4-nNos^−/−^ mice showed increased latency to find the hidden platform and a decreased number of platform crossings during the testing period, suggesting impaired spatial working memory (Fig. 6I and J). Notably, motor ability and skill learning assessed through the rotarod test were normal in Erbb4-nNos^−/−^ mice compared to control mice (Fig. 6K). These results collectively suggest that the genetic deletion of nNos from Erbb4-positive neurons contributes to schizophrenia-relevant behavioral deficits.

Moreover, to investigate the potential impact of nNos genetic deletion from Erbb4-positive neurons on anxiety or depression phenotype, we performed the elevated plus maze test and sucrose preference test, respectively. Figure 6L-O depict the results, revealing no significant differences in time spent in open arms, the number of open arm entries, and the number of total entries in the elevated plus maze test. Additionally, no distinctions were observed between Erbb4-nNos^−/−^ mice and control mice in the sucrose preference test (Fig. 6P). These findings collectively indicate that the downregulation of nNos does not induce an anxiety or depression-like phenotype.

### Behavioral deficits in Erbb4-nNos^−/−^ mice are relieved by clozapine

**Fig. 7:**
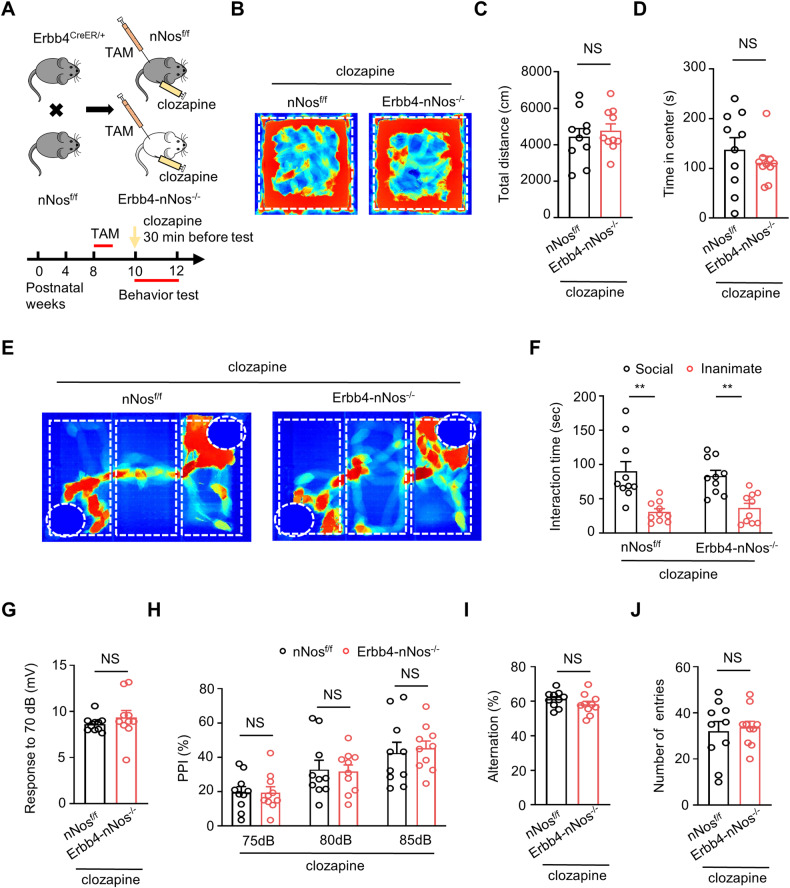
Behavioral deficits in Erbb4-nNos^−/−^ mice are relieved by clozapine. **A** Scheme of experimental design. Top: Breeding diagram for the generation of Erbb4^CreER/+^; nNos^f/f^ mice (Erbb4-nNos^−/−^ mice). Bottom: Time frame of Tamoxifen injection, clozapine injection and behavior testing. **B**–**D** Representative activity tracking **B**, total distance **C** and time in center **D** from nNos^f/f^ and Erbb4-nNos^−/−^ mice after clozapine injection in open field test. **C** Unpaired t test, *t* = 0.5883, *P* = 0.5637. **D** Unpaired t test, *t* = 0.9336, *P* = 0.3629. **E**, **F** Representative activity tracking **E** and interaction time **F** in social interaction test. Two-way ANOVA with Bonferroni’s multiple comparisons test. nNos^f/f^ mice: *P* = 0.0001; Erbb4-nNos^−/−^ mice: *P* = 0.0020. **G**, **H** Quantification of response to 70 dB **G** and percentage of PPI **H** in prepulse inhibition test. **G** unpaired t test, *t* = 0.8817, *P* = 0.3895. **H** Two-way Repeated Measures ANOVA with Bonferroni’s multiple comparisons test, Genotype *F* (1, 18) = 0.006084, *P* = 0.9387. 75 dB, 80 dB, 85 dB: *P* > 0.9999. (**I**, **J** Percentage of spontaneous alternation **I** and the number of arm entries **J** in Y maze. **I** Unpaired t test, *t* = 1.289, *P* = 0.2136. **J** Unpaired t test, *t* = 0.3253, *P* = 0.7487. **B**–**J** *n* = 10 mice for each group. Data are mean ± SEM. NS not significant, *P < 0.05, **P < 0.01.

To further assess whether the aberrant behavior resulting from the genetic deletion of nNos from Erbb4-positive neurons is relevant to schizophrenia, we administrated clozapine (a second-generation antipsychotic medication) to both Erbb4-nNos^−/−^ mice and control mice. All mice received clozapine pretreatment (1 mg/kg i.p.) 30 minutes before the behavioral tests (Fig. 7A). As shown in Fig. 7B-D, there has no difference in travel distance and time in center area between clozapine-treated Erbb4-nNos^−/−^ mice and control mice in the open field test. These results suggest that locomotor hyperactivity in Erbb4-nNos^−/−^ mice is reversed by clozapine treatment. In the three-chamber social test, both Erbb4-nNos^−/−^ mice and control mice spent more time around the ‘social’ cylinder containing the stimulus mouse (Fig. 7E and F), indicating similar social abilities between the two groups. As expected, impaired PPI was restored in mutant mice by the administration of clozapine (Fig. 7G and H). In the Y maze, a test for working memory, the percentage of alternation and the number of entries to arms were similar in clozapine-treated Erbb4-nNos^−/−^ mice and control mice (Fig. 7I and J), indicating that clozapine improves working memory in nNos knockout mice. These results collectively suggest that clozapine ameliorates behavioral deficits in Erbb4-nNos^−/−^ mice, addressing schizophrenia-relevant phenotypes in the open field, the social preference test, PPI, and the Y maze. This further underscores the relevance of nNos deletion in Erbb4-positive neurons to schizophrenia-like behaviors and highlights the potential therapeutic effects of clozapine in mitigating these deficits.

## Discussion

This study elucidates the significance of nNos in Erbb4-positive neurons for GABAergic transmission via a presynaptic mechanism. Furthermore, nNos activity is revealed to be modulated by the NRG1-ErbB4 signaling pathway. Additionally, the downregulation of nNos in the hippocampus induces increased cell-intrinsic excitability of pyramidal neurons and schizophrenia-relevant behavioral manifestations. The current study demonstrates a regulatory mechanism of nNos activity in Erbb4-positive neurons, linking it with presynaptic GABA release, synaptic E/I imbalance onto pyramidal neurons, and behavioral abnormalities relevant to neuropsychiatric phenotypes.

Considerable evidence has demonstrated that nNOS activity can be regulated by calcium (Ca^2+^)-calmodulin (CaM) signaling pathway [41]. Glutamate release from presynaptic terminals activates NMDA receptors, resulting in Ca^2+^ influx. The ensuing binding of Ca^2+^ to CaM, facilitated through PSD95, activates nNOS and induces the production of NO [1]. Additionally, chaperon proteins like heat shock protein (HSP) and CAPON (protein carboxy-terminal PDZ ligand of nNOS), as well as neuronal phosphorylation, have been identified as regulators of nNOS activity [1]. Our findings support the idea that neural activity modulates nNOS activity through diverse mechanisms. Similarly, neural activity has been shown to regulate NRG1 expression and release [27], with ErbB4 and nNOS selectively co-expressed in GABAergic interneurons [25]. In our study, we observed the activation of nNOS by NRG1 through the ErbB4-PI3K-AKT signaling pathway in hippocampal slices. These results uncover a regulatory mechanism elucidating how neural activity influences nNOS activity in GABAergic interneurons.

Neural activities can modulate the strength of GABA release by directly acting on the neurotransmitter-release machinery [42–44]. However, the specific signaling pathways through which neural activities regulate GABAergic release remain incompletely addressed. Considering that the NRG1-ErbB4 signaling pathway promotes GABA release through presynaptic terminals [18, 22], our findings highlight a presynaptic mechanism governing GABA release. The specific mechanism by which nNOS activation enhances presynaptic GABA release is currently unknown. Existing evidence suggests that nNOS/NO signaling may play a role in inhibitory synaptic transmission. A previous study demonstrated NRG1-induced upregulation of nNOS expression in rat cerebellums through the ErbB4 receptor [45]. Importantly, NO has been shown to modulate GABA release or inhibitory postsynaptic currents (IPSC) in cultured cortical neurons, hypothalamic slices, and the CA1 region in vivo [46–48]. The primary signaling mechanisms for NO involve the cGMP pathway through the activation of the canonical NO receptor, soluble guanylate cyclase (sGC), and direct chemical modification of proteins via S-nitrosylation [12]. Previous research has indicated that NO-cGMP signaling participates in hippocampal GABAergic inhibition modulated by NMDARs in an activity-dependent manner [49]. Additionally, some studies propose that certain presynaptic ion channels activated by nNOS may be involved in GABA release. NO increases the GABAergic spontaneous postsynaptic current frequency in amacrine cells in the inner retina, relying on a voltage-independent Ca^2+^ influx pathway, the transient receptor potential canonical (TRPC) channels TRPC5 [12, 13]. Notably, NO can directly activate TRPC channels via S-nitrosylation [50]. However, the specific ion channel and the underlying mechanism require further clarification. In summary, the downstream signaling pathways regulating GABA release by activated nNOS in Erbb4-positive neurons remain elusive, necessitating additional studies to address this issue.

Deficits in GABAergic transmission has been implicated as an underlying mechanism in several neuropsychiatric diseases, including schizophrenia, autistic spectrum disorders, and epilepsy [51–54]. In line with these findings, our recent study reported a reduction of GABAergic transmission in mice exhibiting schizophrenia-relevant behavior phenotypes induced by maternal immune activation [55]. GABA, as the chief inhibitory neurotransmitter in the mature mammalian brain, is crucial for maintaining homeostasis in the neural network [56]. Deficient GABA release has been specifically linked to disturbances in E/I balance [57–59], strongly associated with the pathological manifestations of schizophrenia [60].

Both Erbb4 and nNos have been implicated in GABAergic transmission and abnormal behaviors in rodents. Genetically modified ErbB4 in mice induces impaired brain circuit wiring, leading to recapitulated schizophrenia-related phenotypes, including GABAergic transmission deficits, hyperactivity, decreased PPI, dysfunction of social interaction, impaired working memory, and commanding top-down attention in rodents [17, 20, 21]. Mice with ablation of nNos (nNos^−/−^) exhibit increased locomotor activity, impaired PPI, and impairment of spatial learning and memory [37, 61], all associated with schizophrenia symptoms.

Loss-of-function mutations of both Erbb4 and nNos have been implicated in the pathophysiology of schizophrenia [62–64]. The intracellular kinase domain deletion of Erbb4 has been found in schizophrenia patients, and molecular pathway analysis of structural variants strongly implicates NO signaling in schizophrenia [65, 66]. This study suggests that enhancing ErbB4-nNOS signaling might alleviate GABAergic dysfunction in schizophrenia. It is worth noting that drugs directly targeting nNOS may cause side-effects, such as memory deficits and aggressive behaviors [67]. Further studies are required to develop drugs that specifically target nNOS in NRG1-ErbB4 signaling pathways to treat schizophrenia. In sum, our results demonstrate that nNos deletion in Erbb4-positive neurons reduces GABA release and leads to schizophrenia-relevant behavioral deficits in adult mice. These results may provide insight into the pathophysiological mechanisms of neuropsychiatric disorders.

## Materials and Methods

### Animals

Detailed information regarding the mice used in this study is provided in Supplementary table 1. The Erbb4-reporter mice (Erbb4-td) were generated by crossing Erbb4^CreER/+^ mice with Rosa26^LSL-td/+^ mice (Ai 14), as outlined in our previous study [16]. nNos null mutant mice (nNos^−/−^ mice) were obtained from Jackson Laboratory (JAX# 002986), while Floxed nNos (nNos^f/f^) mice were generously provided by Dr. Jennifer S. Pollock [68]. To create conditional mutant mice with selective deletion of nNOS in Erbb4-positive neurons, nNos^f/f^ mice were crossed with Erbb4^CreER/+^ mice expressing tamoxifen-inducible Cre under the control of the Erbb4 gene. This crossing resulted in the generation of Erbb4^CreER/+^; nNos^f/f^ (Erbb4-nNos^−/−^) mice, with nNos^f/f^ littermates serving as controls. For fluorescent labeling, Erbb4-nNos^−/−^ mice were further crossed with Rosa26^LSL-td/+^ mice to obtain Erbb4-nNos^−/−^-td mice, with Erbb4-td mice serving as controls. Supplementary table 2 provides information on mouse strains and genotyping primers for different mouse lines. C57BL/6 male mice were procured from the Laboratory Animal Center of Sun Yat-sen University. All mice used in this study were maintained on a C57BL/6 background, housed in a 12-hour light/dark cycle (lights on at 7:00), and provided with ad libitum access to food and water. Male mice aged between 8 and 12 weeks were utilized for electrophysiology, immunohistochemistry, and western blots, unless stated otherwise.

### Reagents

Detailed information about antibodies, drugs, chemicals, and recombinant viruses can be found in Supplementary table 3.

### Tamoxifen administration

Tamoxifen administration followed our previous protocols with modifications [16]. Tamoxifen (T5648, Sigma-Aldrich, St. Louis, MO, USA) was dissolved in corn oil (C8267, Sigma-Aldrich, St. Louis, MO, USA) at 20 mg/ml. Eight-week-old mice were injected daily with tamoxifen (100 mg/kg/day, i.p.) for five consecutive days.

### Detection of NO production in cultured hippocampal neurons

Hippocampal neurons were cultured as described in our previous study [69]. Briefly, hippocampal tissue prepared from embryonic day 16 (E16) C57BL/6 mice was digested with 0.25% trypsin for 30 min at 37 °C followed by trituration with a pipette in plating medium (DMEM/F-12 supplemented with 2% N2 and 10% fetal bovine serum). Dissociated neurons were plated onto poly-L-lysine–coated cover slips in 6-well plates at a density of 2.5 × 10^5^ per well for 4 h before replacing the medium with maintenance medium (neural basal medium supplemented with 2% B27). Half the volume of the medium was replaced with fresh medium every 3 days.

The measurement of NO production was conducted utilizing the NO Analyzer (Sievers 280i, Boulder, CO) as detailed previously [70]. Neurons cultured for 14 days in vitro (DIV) were employed in the experiments. Cell culture media from neurons treated with vehicle or NRG1 (5 nM for 20 min) were collected. Subsequently, medium aliquots were refluxed in glacial acetic acid containing sodium iodide in the reaction chamber. Under these conditions, NO_2_^−^ is quantitatively reduced to NO, which was then quantified by a chemiluminescence detector after reacting with ozone. The amount of NO was calculated using the standard curve of sodium nitrite.

### Immunofluorescence staining

Immunofluorescence staining of frozen sections was conducted following the methodology outlined in our prior study [71]. Briefly, mice were anesthetized, and their brains were transcardially perfused with 4% paraformaldehyde (PFA). The extracted brains were fixed in 4% PFA overnight and subsequently equilibrated in 30% sucrose at 4 °C. Free-floating coronal sections, 40-μm-thick, of the entire hippocampus were obtained using a freezing microtome (Leica SM2000R, Heidelberg, Germany). Specimens were blocked in a solution comprising 1% BSA, 10% normal goat serum, and 0.25% Triton X-100 (Sigma) at 37 °C for 1 h. Following this, sections were stained with primary antibodies at 37 °C for 2 h, followed by an overnight incubation at 4 °C. The subsequent day, sections were stained with secondary antibodies at 37 °C for 2 h. Finally, slices were mounted onto slides, and images were acquired with a confocal laser-scanning microscope (Nikon A1, Tokyo, Japan).

### Western blot analysis

For Western blot analysis, the examination of total protein and phosphorylated protein levels in the hippocampus was carried out through western blot analysis [39]. Briefly, hippocampal tissues were homogenized in ice-cold RIPA lysis buffer (50 mM Tris-HCl, pH 7.4, 150 mM NaCl, 2 mM EDTA, 1% sodium deoxycholate, 1% SDS, 1 mM PMSF, 50 mM sodium fluoride, 1 mM sodium vanadate, 1 mM DTT) in the presence of protease inhibitors (1 mM PMSF, protease inhibitor cocktail, phosphatase inhibitor cocktail 2). After incubation on ice for 30 min, the homogenates were centrifuged at 12,000 rpm (15 min, 4 °C). The total protein concentration was quantified using an Enhanced BCA Protein Assay Kit (Beyotime, P0012) and adjusted to 4.5 mg/ml. After being boiled in 5×SDS–PAGE Sample Loading Buffer, 45 μg proteins were subjected to electrophoresis (10% SDS/PAGE gels), followed by electroblotting onto presoaked PVDF membranes (BioRad). The blots were blocked in 5% no-fat milk in PBST (100 nM phosphate buffer, pH 7.5, containing 150 nM NaCl and 0.1% Tween-20) at room temperature for 1 h. Then all blots were incubated in primary antibodies overnight at 4 °C and switched to the HRP-conjugated secondary goat anti-rabbit at 37 °C for 1 h. Protein bands were visualized using a chemiluminescence system (ChemiDoc^TM^ XRS^+^, BioRad), and the protein expressions were semi-quantitatively evaluated using Image J software (NIH).

### Slice preparation

Hippocampal slices were meticulously prepared following the methodology outlined in our prior study [39]. In brief, mice were deeply anesthetized with pentobarbital (100 mg/kg i.p.) and transcardially perfused with a 4 °C slice-cutting solution comprising (in mM): 220 sucrose, 2.5 KCl, 1.3 CaCl_2_-2H_2_O, 2.5 MgSO_4_, 1 NaH_2_PO_4_-2H_2_O, 26 NaHCO_3_, and 10 D-glucose, aimed at safeguarding neurons and preserving the functional connectivity of brain slices. Subsequently, mice were decapitated, and brains promptly extracted and kept in ice-cold cutting solution. Transverse hippocampal slices (300 μm thickness) were precisely sectioned by a VT1200S vibratome (Leica, Germany) in ice-cold cutting solution. The slices were then incubated in regular artificial cerebrospinal fluid (ACSF) containing (in mM): 126 NaCl, 3 KCl, 1.2 NaH_2_PO_4_-2H_2_O, 1 MgSO_4_, 2.0 CaCl_2_-2H_2_O, 26 NaHCO_3_ and 10 D-glucose for 30 minutes at 33 °C and 1 hour at room temperature (25 ± 1 °C) before recording. All solutions underwent oxygenation with 95% O_2_ and 5% CO_2_.

### Whole-cell patch clamp recordings

Electrophysiological recordings were conducted according to the procedures delineated in our earlier studies [18, 71]. Slices were placed in the recording chamber and perfused (3 ml/min) with oxygenated regular ACSF at 32–34 °C for patch clamp recordings. In extracellular Ca^2+^-free experiment, 2 mM EGTA was added in modified ACSF instead of CaCl_2_-2H_2_O. Whole-cell patch-clamp recordings from CA1 neurons were visualized with infrared optics using an upright microscope (Eclipse FN1, Nikon, Japan) equipped with a 40× water-immersion lens (N40X-NIR, Nikon, Japan) and Digital CMOS camera (C11440-42U, Hamamatsu, Japan). The patch pipettes (4–6 MΩ) were pulled from borosilicate glass with filament (BF150-86-10, Sutter Instruments, USA) using a flaming/brown micropipette puller (P-97, Sutter Instruments, USA).

To record evoked inhibitory postsynaptic currents (eIPSCs), axons in the stratum radiatum were stimulated with a two-concentric bipolar stimulating electrode (CBARC75, FHC, USA) connected to a stimulus isolation unit (ISO-Flex Stimulus Isolator; A.M.P.I.) at a frequency of 0.033 Hz. Pipettes were filled with internal solution (in mM): 100 CsCH_3_SO_3_, 60 CsCl, 10 HEPES, 0.2 EGTA, 1 MgCl_2_, 4 ATP-Mg, 0.3 GTP-Na, and 5 QX-314 (pH 7.25, 280 mOsm). To measure the paired pulse ratio (PPR) of eIPSCs in Fig. 4, four continuous pulses were applied at an interval of 50 ms. In Figure S2, PPR of eIPSCs was measured with paired stimulation at different intervals, including 25 ms, 50 ms, 100 ms and 200 ms.

For miniature IPSCs (mIPSCs) recording, 1 µM TTX was added to ACSF, the concentration of CsCl was increased to 140 mM and CsCH_3_SO_3_ was omitted in the pipette solution. Both mIPSCs and eIPSCs were pharmacologically isolated in the presence of DL-AP5 (100 µM) and CNQX (20 µM) at the holding potential of -70 mV and were verified by the addition of 20 μM Bicuculline methiodide (BMI). In some experiments, hippocampal slices were treated with inhibitors (300 μM L-NAME, 200 nM L-NPA or 40 µM Vigabatrin) prior to the application of 5 nM NRG1.

To record miniature excitatory postsynaptic currents (mEPSCs), we added 1 µM TTX and 20 µM BMI to ACSF. Pipettes were filled with a K^+^-based solution containing (in mM) 105 K-gluconate, 30 KCl, 10 HEPES, 10 phosphocreatine, 4 ATP-Mg, 0.3 GTP-Na and 0.3 EGTA (pH 7.35, 285 mOsm). For recording action potentials (APs), we used K^+^-based intracellular solution. To characterize the membrane and firing properties of neurons, we applied hyperpolarizing and depolarizing current steps (-200 pA to 350 pA) for 500 ms at 0.2 Hz in current-clamp configuration.

To record spontaneous excitatory postsynaptic currents and inhibitory postsynaptic currents (sEPSCs/sIPSCs), patch electrodes were filled with a solution containing the following (in mM): 125 CsCH_3_SO_3_, 5 CsCl, 10 HEPES, 0.2 EGTA, 1 MgCl2, 4 Mg-ATP, 0.3 Na-GTP, 10 phosphocreatine, 5 QX-314 (pH 7.30, 280 mOsm). For sEPSC recordings, voltage clamp recordings were performed at −60 mV, and sIPSC recordings were measured at +10 mV.

Data were recorded using a Multiclamp 700B amplifier and a Digidata 1550 A (Molecular Devices, USA), digitized at 10 kHz and filtered at 1 kHz. We collected data when the series resistance fluctuated within 20% of initial values and analyzed it using pClamp 10.7 software (Molecular Devices, USA).

### Behavioral analysis

Open field test, pre-pulse inhibition, social interaction test, Morris water maze and rotarod test were performed as described in our previous study [39, 55]. Y maze was performed as previously described [72].

### Open field test

Mice were placed in the center of the chamber (40 × 40 × 40 cm) located in a soundproof box and allowed to explore for 30 min. Motor activity was recorded with an infrared camera placed above the box. The total distance, time in the center and central distance during 30 min was measured (Jiliang Software Technology, Shanghai, China). The arena was cleaned with 75% ethanol and dried thoroughly after each test session.

### Prepulse inhibition (PPI)

PPI tests were conducted using a SR-Lab System apparatus (San Diego Instruments, San Diego, CA, USA). Mice were habituated to the chamber with a 70-dB background white noise for 5 min. During the test, mice were subjected to 12 startle trials (20 ms, 120 dB) and 12 prepulse/startle trials (20 ms white noise at 75, 80, or 85 dB at 100-ms intervals and 20 ms 120-dB startle stimulus), performed pseudo-randomly. PPI (%) was calculated according to the formula: 100 × (startle amplitude for pulse alone − startle amplitude for the pulse with prepulse) / startle amplitude for pulse alone. The chamber was cleaned with 75% ethanol and dried thoroughly after each test session.

### Social interaction test

Adult male mice underwent a social interaction test within a blue Plexiglas rectangular box (60 × 40 × 30 cm) comprising three interconnected chambers. Each end chamber housed a clear Plexiglas cylinder, with one designated as the “social” cylinder containing a stimulus mouse (adult wild-type male mice unfamiliar to the test mice), while the other served as the “non-social” cylinder, left empty. Initially, experimental mice were placed in the center chamber and allowed 5 minutes to freely explore the chambers without cylinders. Subsequently, the same procedure was repeated, but with two empty cylinders in the chambers, extending the exploration time to 10 minutes. In the final phase, mice were granted an additional 10 minutes to explore the chambers containing a “social” and a “non-social” cylinder. The sessions were recorded, and the time spent around each cylinder was analyzed using the tracking system (Jiliang Software Technology, Shanghai, China). Post-testing, the box and cylinders underwent cleaning with 75% ethanol, followed by thorough drying after each session.

### Morris water maze

The experiment took place in a circular pool crafted from blue plastic (diameter, 120 cm), enclosed by white curtains from the pool’s edge to the ceiling. The pool, filled with water (20–22 °C) made opaque by adding nontoxic white paint, featured a concealed Plexiglas platform (diameter, 10 cm) positioned 1 cm below the water surface. Mice underwent a 4-day training regimen with 4 trials per day, each lasting 60 seconds. Four different start positions were employed to ensure the utilization of visual-spatial memory by the mice to locate the hidden platform. On the fourth day, a 60-second probe trial was conducted, during which the platform was removed, and the mice were placed into the pool, scored for the number of platform crossings. Escape latency and the number of platform crossings were analyzed using the tracking system (Jiliang Software Technology, Shanghai, China). On the fifth day, mice were assessed for their ability to locate a visible platform within 60 seconds, with any mouse experiencing two 60-second trials being eliminated from the study.

### Y maze

The Y-maze apparatus, featuring three identical arms (25 × 10 × 25 cm) positioned at 120° angles to each other, formed a Y shape. Arms A, B, and C were the designated labels for the respective arms. Mice were introduced at the terminus of arm A and allowed to freely explore the maze for 5 minutes. Limb positioning within an arm was considered an arm entry. An alternation occurred when mice sequentially explored all three arms. The tracking system (Jiliang Software Technology, Shanghai, China) recorded and analyzed mouse activity and spontaneous behavioral alternations. The percentage (%) of spontaneous alternation behavior was calculated as follows: % alternation = ([number of alternations] / [total number of arm entries – 2]) × 100. Post each test session, the apparatus underwent cleaning with 75% ethanol and thorough drying.

### Rotarod test

Motor coordination and balance were assessed using an accelerating rotarod. Experimental mice underwent evaluation of their balance on a rotating bar that accelerated from 4 to 40 rpm over a 5-minute period. The recording system (Jiliang Software Technology, Shanghai, China) measured the latency to fall from the rod. Mice received two trials per day for two consecutive days with a 30-minute interval. The rod underwent cleaning with 75% ethanol and thorough drying after each test session.

### Elevated plus maze

The test, as previously described [73], utilized an apparatus comprising two open arms (30 × 5 cm), two enclosed arms (30 × 5 × 15 cm), and a central platform (5 × 5 cm) at the intersection of the four arms. Each mouse, placed in the central platform facing one open arm, explored the apparatus for 5 minutes. The recording system (Jiliang Software Technology, Shanghai, China) measured time spent in open arms, the number of open arm entries, and total entries. The apparatus was cleaned with 75% ethanol and dried thoroughly after each test session.

### Sucrose preference test

This test, following a two-bottle choice procedure [74], involved presenting each mouse with two drinking bottles: 1% (w/v) sucrose and water, respectively. Before the test, mice were individually housed and acclimated to 1% (w/v) sucrose for 24 hours. Subsequently, mice were water-deprived with free access to food for 16 hours. On the test day, each mouse had access to two pre-weighed bottles, one containing water and the other containing 2% (w/v) sucrose. After 24 hours of consumption, the bottles were reweighed, and the preference for sucrose over water was calculated as sucrose / (sucrose + water) × 100%.

### Statistical analysis

Statistical analyses were conducted using SPSS version 21.0 (SPSS, Inc., Chicago, IL, USA) or GraphPad Prism 7 (GraphPad Software, Inc., San Diego, CA, USA). The presentation of all data adhered to the format of means ± SEM. Comparison of data between two groups was performed using a paired or unpaired Student’s *t*-test, while results involving more than two parameters were subjected to a one-way or two-way ANOVA. The number of animals, recorded neurons or independent experiments is provided in figure legends. The determination of sample size was based on previous studies [18, 39], and animals were randomly assigned to experimental groups. There were no animal exclusion criteria. Throughout electrophysiological experiments, behavioral tests, and analysis, investigators remained blind to both mouse genotype and treatment group. All tests were two-sided, and statistical significance was considered at *P* < 0.05.

### Supplementary Materials

Supplementary figures (S1-S6) are available in *Supplementary Materials*. Further details on mouse strains and genotyping primers for different mouse lines can be found in Supplementary Tables 1-2. Comprehensive information regarding antibodies, drugs, chemicals, and recombinant viruses is presented in Supplementary Table 3.

### Supplementary information

Supplemental Material-Figures and tables

checklist

Supplemental Material-western blots

## Data Availability

All data are available in the main text or the supplementary materials.
