# Supplementary material for: nNOS in Erbb4-positive neurons regulates GABAergic transmission in mouse hippocampus
Source: Cell Death Dis. 2024 Feb 23;15(2):167. doi: 10.1038/s41419-024-06557-1 (PMC10891175; doi:10.1038/s41419-024-06557-1)
Supplement: Supplementary file 1 — Supplemental Material-Figures and tables [file 41419_2024_6557_MOESM1_ESM.docx]

**Supplementary materials for**

**nNOS in Erbb4-positive neurons regulates GABAergic transmission in mouse hippocampus**

Chaofan Wan^#^, Yucen Xia^#^, Jinglan Yan, Weipeng Lin, Lin Yao, Meng Zhang, Inna Gaisler-Salomon, Lin Mei, Dong-Min Yin*, Yongjun Chen*

This file includes:

1. Supplementary Figures S1 to S6

2. Supplementary Tables 1-3

**1. Supplementary Figure and Figure Legends**


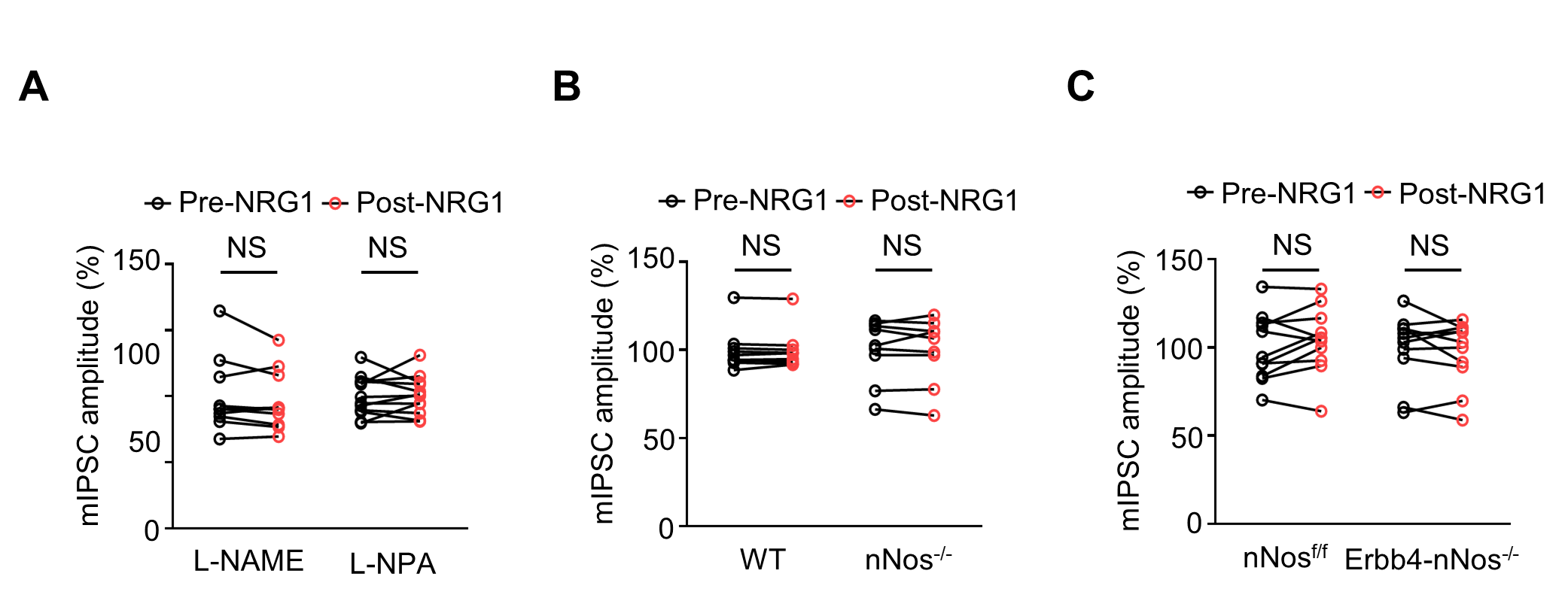


**Figure S1. Application of NRG1 has no effect on mIPSC amplitude. (A)** Quantification of mean values of mIPSC amplitude before and after NRG1 treatment in the presence of 300 μM L-NAME or 200 nM L-NPA. L-NAME, paired *t* test, *t* = 1.237, *P* = 0.2475. n = 10 neurons from 4 mice; L-NPA, paired *t* test, *t* = 0.4749, *P* = 0.6442. n = 12 neurons from 3 mice. **(B)** Quantification of mean values of mIPSC amplitude from WT and nNos^-/-^ mice before and after 5 nM NRG1 treatment. WT: paired *t* test, *t* = 0.1521, *P* = 0.8828; nNos^-/-^: paired *t* test, *t* = 0.1943, *P* = 0.8508. n = 9 neurons from 3 mice for each group. **(C)** Quantification of mean values of mIPSC amplitude from nNos^f/f^ and Erbb4-nNos^-/-^ mice before and after 5 nM NRG1 treatment. nNos^f/f^: paired *t* test, *t* = 1.481, *P* = 0.1693; Erbb4-nNos^-/-^: paired *t* test, *t* = 0.9084, *P* = 0.3851. n = 11 neurons from 5 nNos^f/f^ mice; n = 11 neurons from 6 Erbb4-nNos^-/-^ mice. Data are mean ± SEM. NS, not significant, **P* < 0.05, ***P* < 0.01.


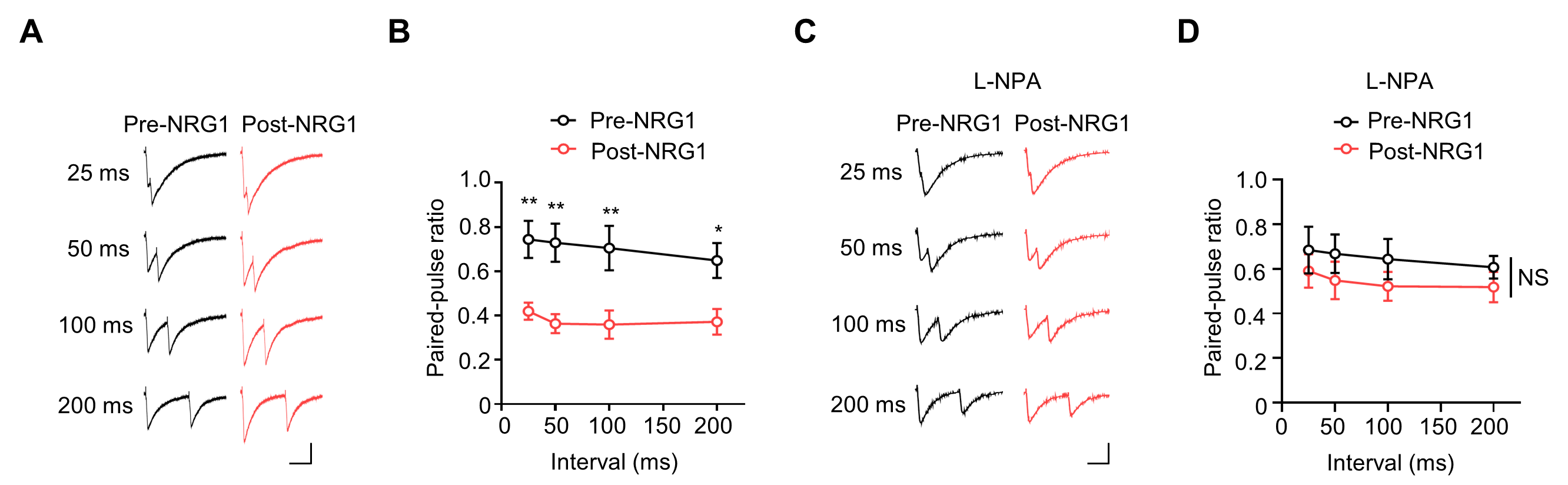


**Figure S2. NRG1 decreases the paired-pulse ratio of eIPSC, that is blocked by application of L-NPA. (A)** Representative traces of PPR of eIPSCs in CA1 pyramidal neurons before and after NRG1 treatment. **(B)** Quantification of mean values of PPR. Scale bar = 100 ms, 200 pA. Two-way Repeated Measures ANOVA with Bonferroni's multiple comparisons test, Group *F* (1, 22) = 14.81, *P* = 0.0009; 25 ms: *P* = 0.0082; 50ms: *P* =0.0023; 100ms: *P* = 0.0043; 200ms: *P* = 0.0321. n = 12 neurons from 3 mice. **(C)** Representative traces of PPR of eIPSCs in CA1 pyramidal neurons before and after NRG1 treatment in the presence of L-NPA in ACSF. **(D)** Quantification of mean values of PPR. Scale bar = 100 ms, 200 pA. Two-way Repeated Measures ANOVA with Bonferroni's multiple comparisons test, Group *F* (1, 22) = 1.535, *P* = 0.2285; 25 ms: *P* > 0.9999; 50ms: *P* > 0.9999; 100ms: *P* > 0.9999; 200ms: *P* > 0.9999. n = 12 neurons from 3 mice. Data are mean ± SEM. NS, not significant, **P* < 0.05, ***P* < 0.01.


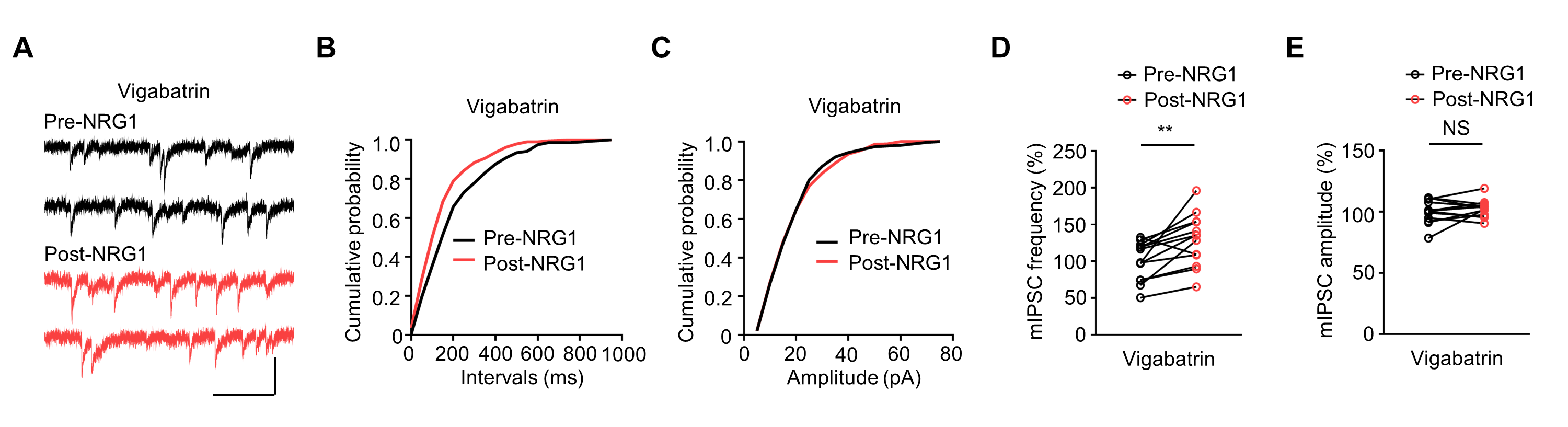


**Figure S3. The increased mIPSC frequency by NRG1 is not blocked by GABA transaminase (GABAT) inhibitor, Vigabatrin. (A)** Representative traces of mIPSCs before and after 5 nM NRG1 treatment in the presence of 40 µM Vigabatrin, in artificial cerebrospinal fluid (ACSF). Scale bar = 250 ms, 50 pA. **(B-C)** Cumulative plots of mIPSC interevent intervals **(B)** and amplitude **(C)**. **(D-E)** Quantification of mean values of mIPSC frequency **(D)** and mIPSC amplitude **(E)**. **(D)** paired *t* test, *t* = 3.640, *P* = 0.0034; **(E)** paired *t* test, *t* = 1.012, *P* = 0.3314. n = 13 neurons from 3 mice. NS, not significant, **P* < 0.05, ***P* < 0.01.


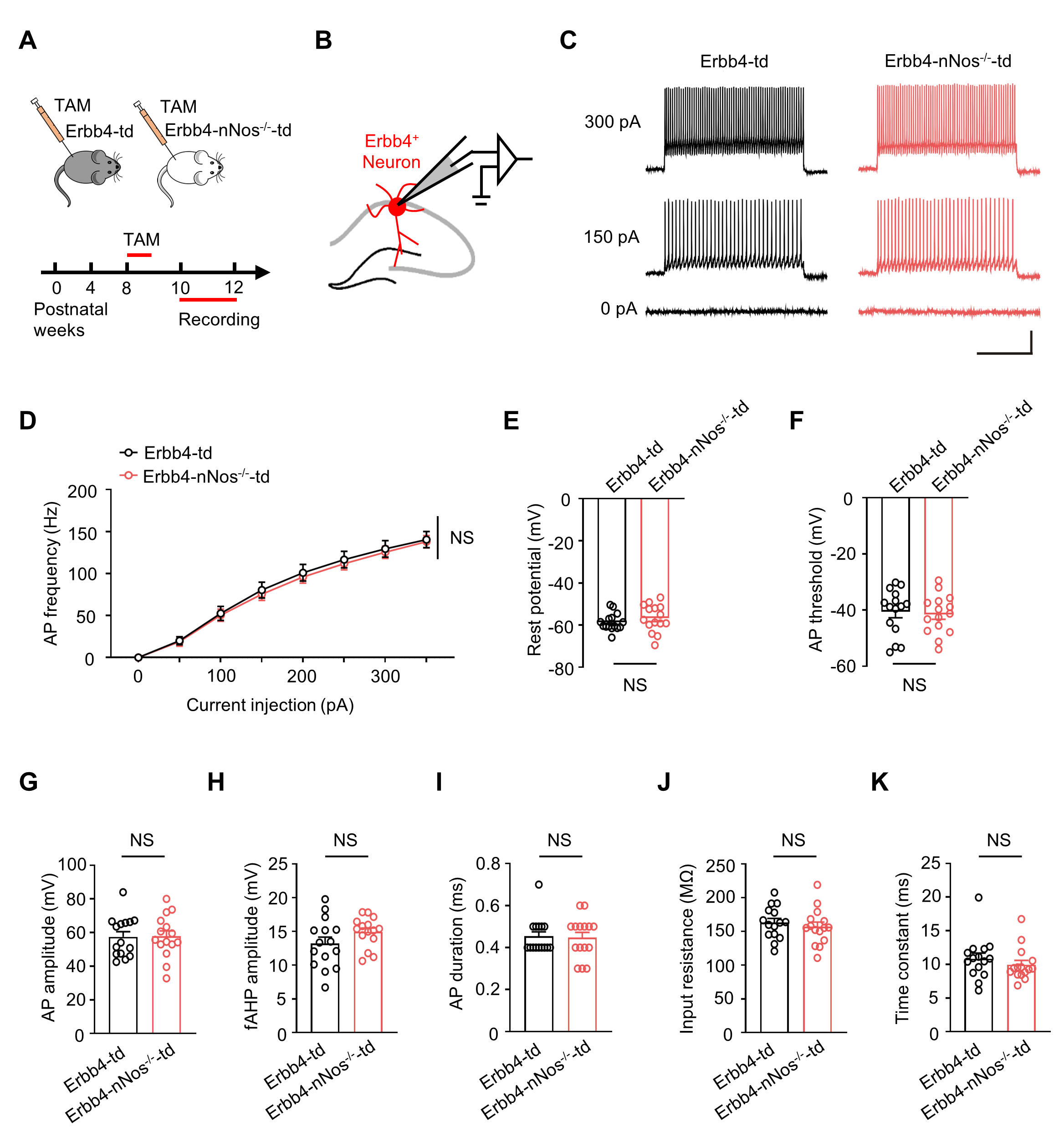


**Figure S4. Genetic deletion of nNos from Erbb4-positive neurons does not influence firing rate of fast-spiking Erbb4^+^ neurons. (A)** Scheme of experimental design and time frame of Tamoxifen injection and electrophysiological recording. **(B)** Schematic of whole-cell recordings from Erbb4 positive neurons in hippocampus. **(C)** Representative traces of APs in CA1 fast-spiking Erbb4 positive neurons from Erbb4-td and Erbb4-nNos^-/-^-td mice. Scale bar = 200 ms, 20 mV. **(D)** Quantification of AP frequency induced by different intensity of current injection from Erbb4-td and Erbb4-nNos^-/-^-td mice. Two-way Repeated Measures ANOVA with Bonferroni's multiple comparisons test, Genotype *F* (1, 28) = 0.1086, *P* = 0.7441. **(E)** Quantification of rest potential. Unpaired t test, *t* = 0.9512, *P* = 0.3496. **(F)** Quantification of AP threshold. Unpaired t test, *t* = 0.3307, *P* = 0.7433. **(G)** Quantification of AP amplitude. Unpaired t test, *t* = 0.07073, *P* = 0.9441. **(H)** Quantification of fAHP amplitude. Unpaired t test, *t* = 1.543, *P* = 0.1340. **(I)** Quantification of AP duration. Unpaired t test, *t* = 0.1994, *P* = 0.8434. **(J)** Quantification of input resistance. Unpaired t test, *t* = 0.6538, *P* = 0.5186. **(K)** Quantification of time constant. Unpaired t test, *t* = 0.8687, *P* = 0.3924. **(D-K)** n = 15 neurons from 3 mice for each group. Data are mean ± SEM. NS, not significant, **P* < 0.05, ***P* < 0.01.


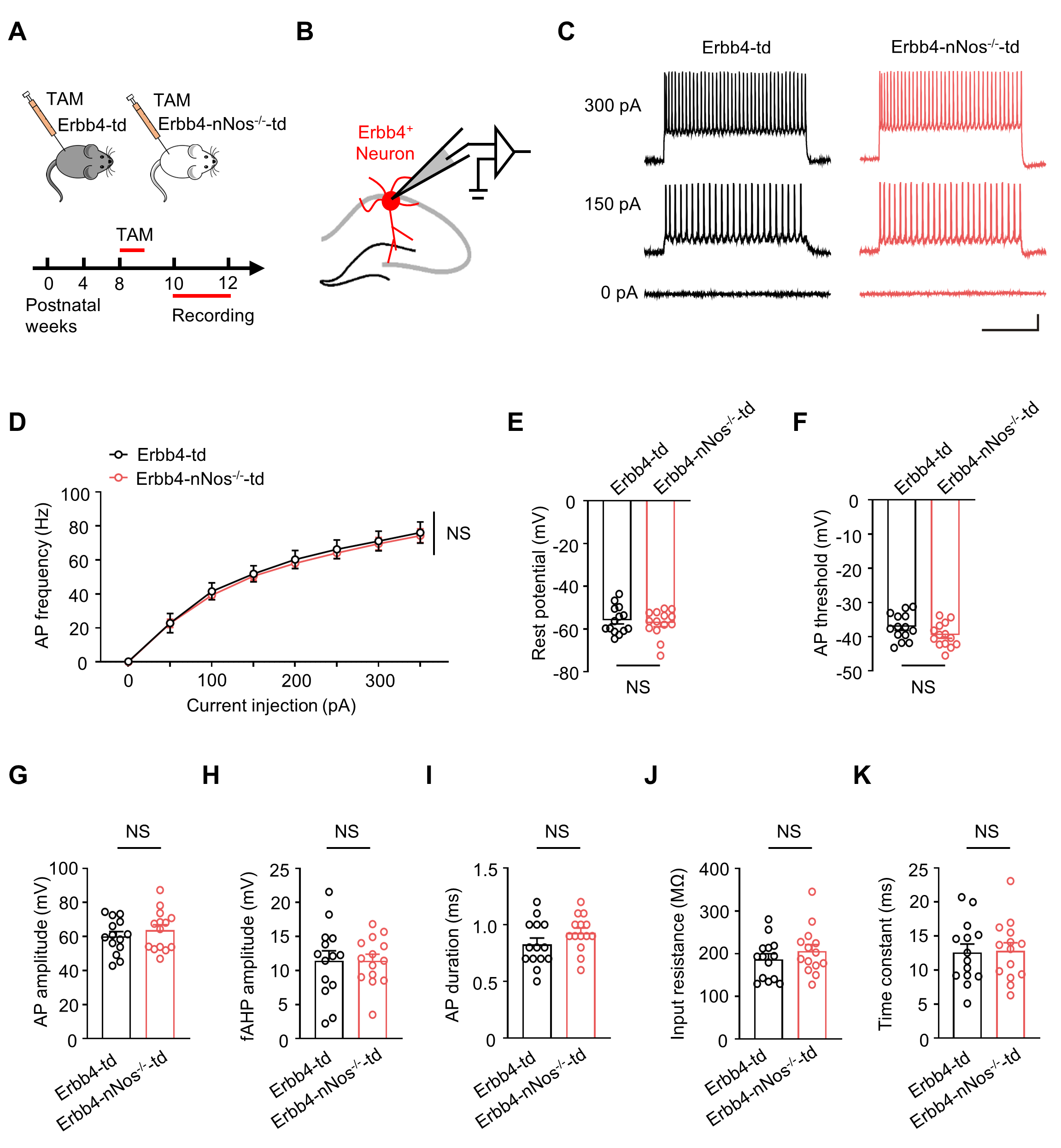


**Figure S5. Genetic deletion of nNos from Erbb4-positive neurons does not influence firing rate of regular-spiking Erbb4+ neurons. (A)** Scheme of experimental design and time frame of Tamoxifen injection and electrophysiological recording. **(B)** Schematic of whole-cell recordings from Erbb4 positive neurons in hippocampus. **(C)** Representative traces of APs in CA1 regular-spiking Erbb4 positive neurons from Erbb4-td and Erbb4-nNos^-/-^-td mice. Scale bar = 200 ms, 20 mV. **(D)** Quantification of AP frequency induced by different intensity of current injection from Erbb4-td and Erbb4-nNos^-/-^-td mice. Two-way Repeated Measures ANOVA with Bonferroni's multiple comparisons test, Genotype *F* (1, 26) = 0.08042, *P* = 0.7790. **(E)** Quantification of rest potential. Unpaired t test, *t* = 0.5630, *P* = 0.5783. **(F)** Quantification of AP threshold. Unpaired t test, *t* = 1.749, *P* = 0.0922. **(G)** Quantification of AP amplitude. Unpaired t test, *t* = 0.8485, *P* = 0.4039. **(H)** Quantification of fAHP amplitude. Unpaired t test, *t* = 0.001248, *P* = 0.9990. **(I)** Quantification of AP duration. Unpaired t test, *t* = 1.457, *P* = 0.1570. **(J)** Quantification of input resistance. Unpaired t test, *t* = 0.9892, *P* = 0.3317. **(K)** Quantification of time constant. Unpaired t test, *t* = 0.1373, *P* = 0.8919. **(D-K)** n = 14 neurons from 3 mice for each group. Data are mean ± SEM. NS, not significant, **P* < 0.05, ***P* < 0.01.


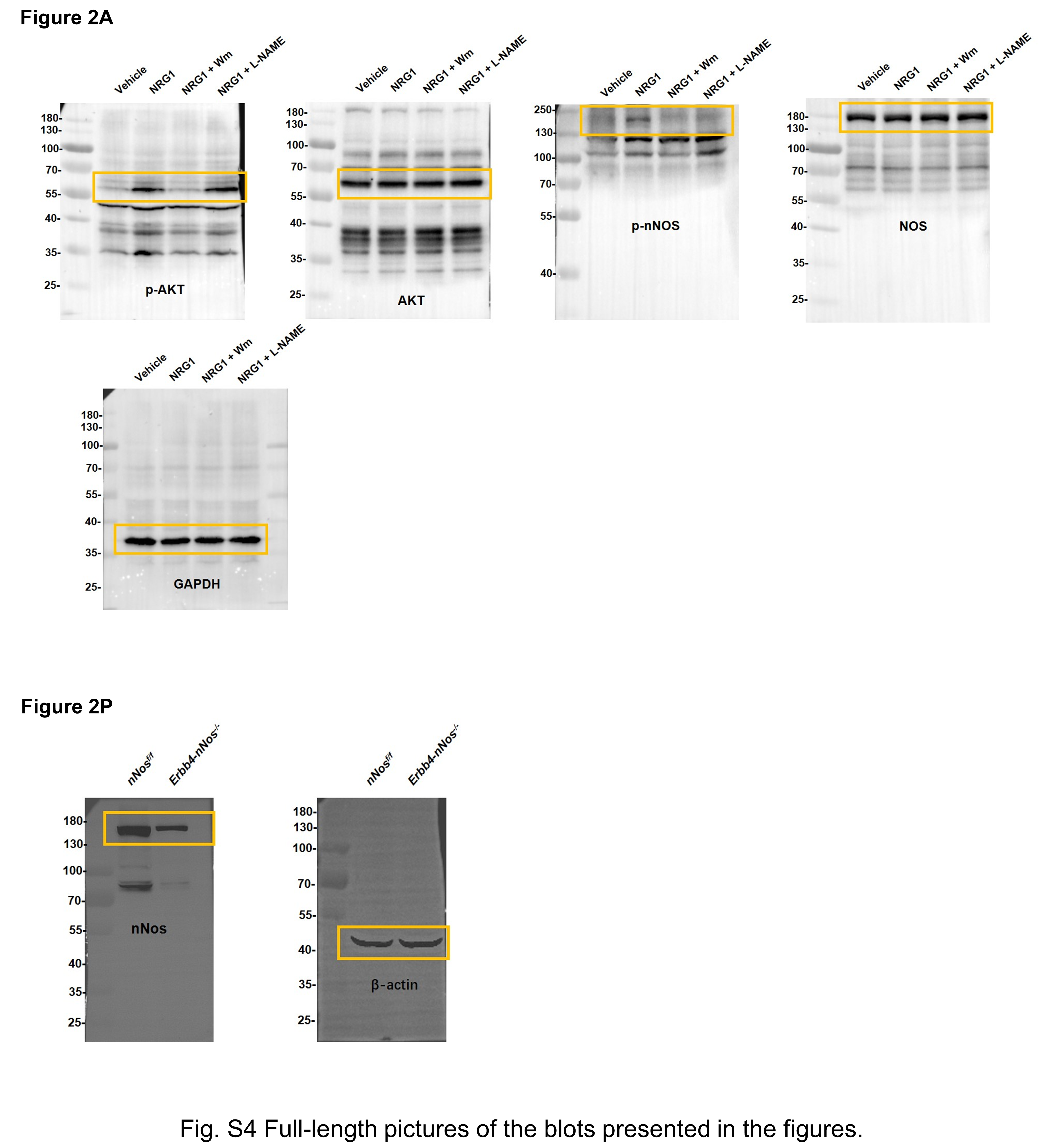


**Figure S6. Full-length pictures of the blots presented in the figures.**

**2. Supplementary tables**

**Supplementary Table 1** Mouse strains

| **Mouse Strains** | **Source** | **Identifier** |
| --- | --- | --- |
| C57BL/6 | The Laboratory Animal Center of Sun Yat-sen University | N/A |
| B6.Cg-Erbb4^tm1.1(cre/ERT2)Aibs^/J (Erbb4^CreER/+^) | The Jackson Lab, USA | Cat# 012360 |
| B6.Cg-Gt(ROSA)26Sor^tm9(CAG-tdTomato)Hze^/J (Rosa26^LSL-td/+^) | The Jackson Lab, USA | Cat# 007909 |
| B6.129S4-Nos1^tm1Plh^/J (nNos^-/-^) | The Jackson Lab, USA | Cat# 002986 |
| nNos^f/f^ | Dr. Jennifer S. Pollock | N/A |

**Supplementary Table 2** Genotyping primers

| **Gene** | **Genotyping primers** |
| --- | --- |
| Erbb4^CreER/+^ | 5’-CCT GCA GGA ATA CAG CAC AA-3’ |
|  | 5’-AAA GAT GGG GCT CTT TGA CA-3’ |
|  | 5’-GGG AGG ATT GGG AAG ACA AT-3’ |
| Rosa26^LSL-td/+^ | 5’-AAG GGA GCT GCA GTG GAG TA-3’ |
|  | 5’- CCG AAA ATC TGT GGG AAG TC-3’ |
|  | 5’-GGC ATT AAA GCA GCG TAT CC-3’ |
|  | 5’- CTG TTC CTG TAC GGC ATG G-3’ |
| nNos^-/-^ | 5’-TCA GAT CTG ATC CGA GGA GG-3’ |
|  | 5’-TTC CAG AGC GCT GTC ATA GC-3’ |
|  | 5’-CTT GGG TGG AGA GGC TAT TC-3’ |
|  | 5’-AGG TGA GAT GAC AGG AGA TC-3’ |
| nNos^f/f^ | 5’-TGT TCC ATG CAC TGT GTT AGC-3’ |
|  | 5’-GAT ACG TGT AGA GGG CAA ATG-3’ |

**Supplementary Table 3** Key resources table

| **REAGENT OR RESOURCE** | **SOURCE** | | **IDENTIFIER** |
| --- | --- | --- | --- |
| **Primary antibodies** |  | |  |
| AKT antibody [N3C2], Internal (WB, 1:4000) | GeneTex | | Cat# GTX121937 |
| Anti-Akt phospho (Ser473) antibody (WB, 1:4000) | | Arigo | Cat# ARG51558 |
| nNOS Polyclonal Antibody (IF, 1:1000; WB, 1:4000) | Thermo Fisher | | Cat# SF244209 |
| Anti-nNOS (neuronal) (phospho S1417) antibody (WB, 1:4000) | Abcam | | Cat# ab5583 |
| beta Actin Antibody (WB, 1:8000) | Abways | | Cat# AB0035 |
| GAPDH Antibody (WB, 1:8000) | Abways | | Cat# AB0037 |
| **Secondary antibodies** |  | |  |
| Goat Anti-Rabbit IgG H&L (HRP) (WB, 1:10000) | Abcam | | Cat# ab6721 |
| Goat Anti-Rabbit IgG H&L (Alexa Fluor® 488) (IF, 1:1000) | Abcam | | Cat# ab150077 |
| **Drugs** |  | |  |
| NRG1 | ProSpec | | Cat# CYT-733 |
| DL-2-Amino-5-phosphonopentanoic acid (DL-AP5) | Tocris Bioscience | | Cat# 0105 |
| 6-Cyano-7-nitroquinoxaline-2, 3-dione (CNQX) | Tocris Bioscience | | Cat# 0190 |
| (-)-Bicuculline methobromide (BMI) | Tocris Bioscience | | Cat# 0109 |
| QX-314 | Tocris Bioscience | | Cat# 1014 |
| Tetrodotoxin (TTX) | Tocris Bioscience | | Cat# 1078 |
| Wortmannin | Tocris Bioscience | | Cat# 1232 |
| Nω-propyl-L-Arginine (L-NPA) | Cayman Chemical | | Cat# 80587 |
| Nω-Nitro-L-arginine methyl ester hydrochloride (L-NAME) | Sigma-Aldrich | | Cat# N5751 |
| Sodium nitroprusside dihydrate (SNP) | Sigma-Aldrich | | Cat# 71778 |
| Tamoxifen | Sigma-Aldrich | | Cat# T5648 |
| Corn oil | Sigma-Aldrich | | Cat# C8267 |
| Vigabatrin | MCE | | Cat# HY-15399 |

**Abbreviates**: **IF**, immunofluorescence; **WB**, western blot.
