# Supplementary figures and images for: nNOS in Erbb4-positive neurons regulates GABAergic transmission in mouse hippocampus
Source: Cell Death Dis. 2024 Feb 23;15(2):167. doi: 10.1038/s41419-024-06557-1 (PMC10891175; doi:10.1038/s41419-024-06557-1)

Figure 2A

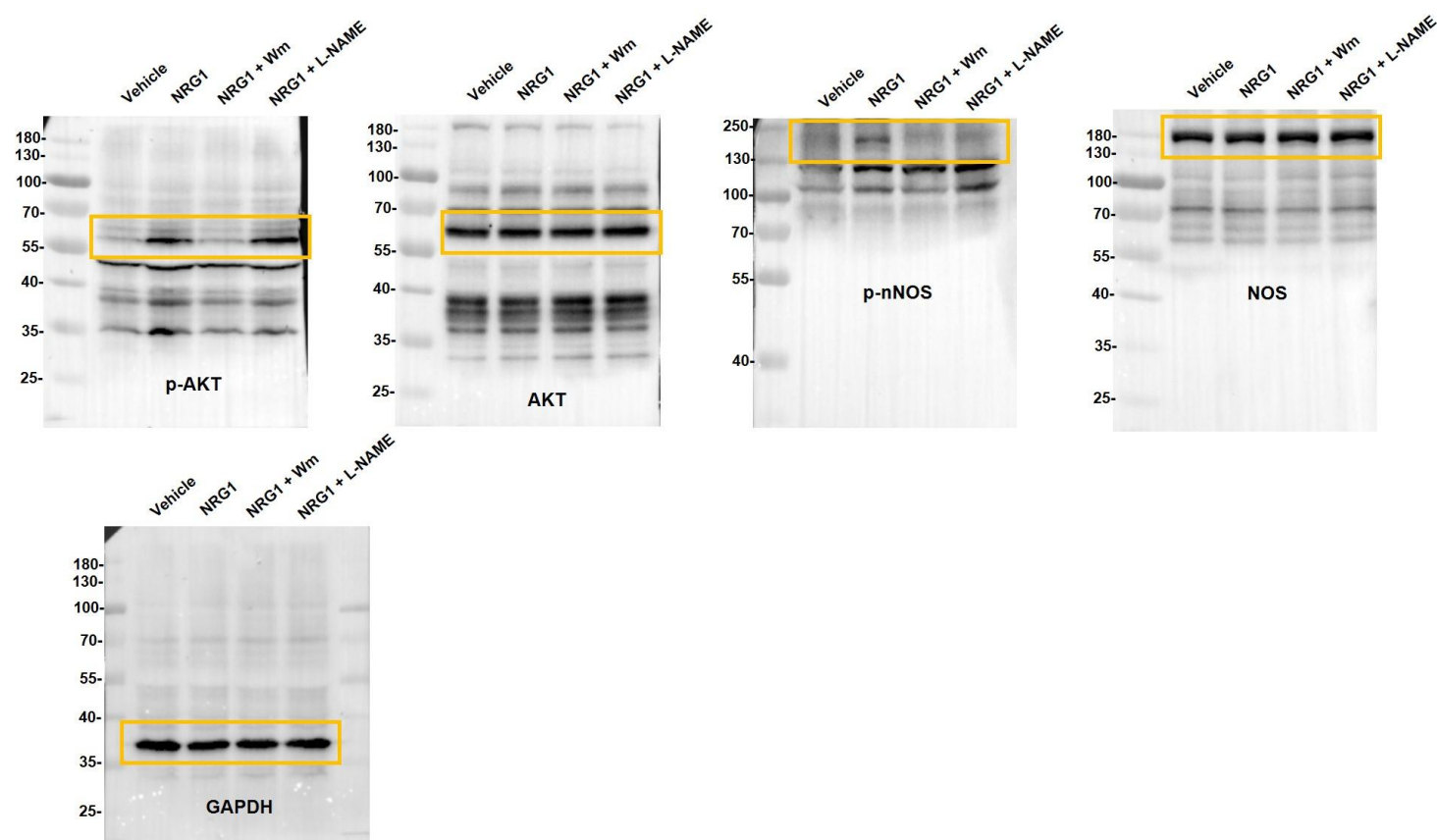

Figure 2P

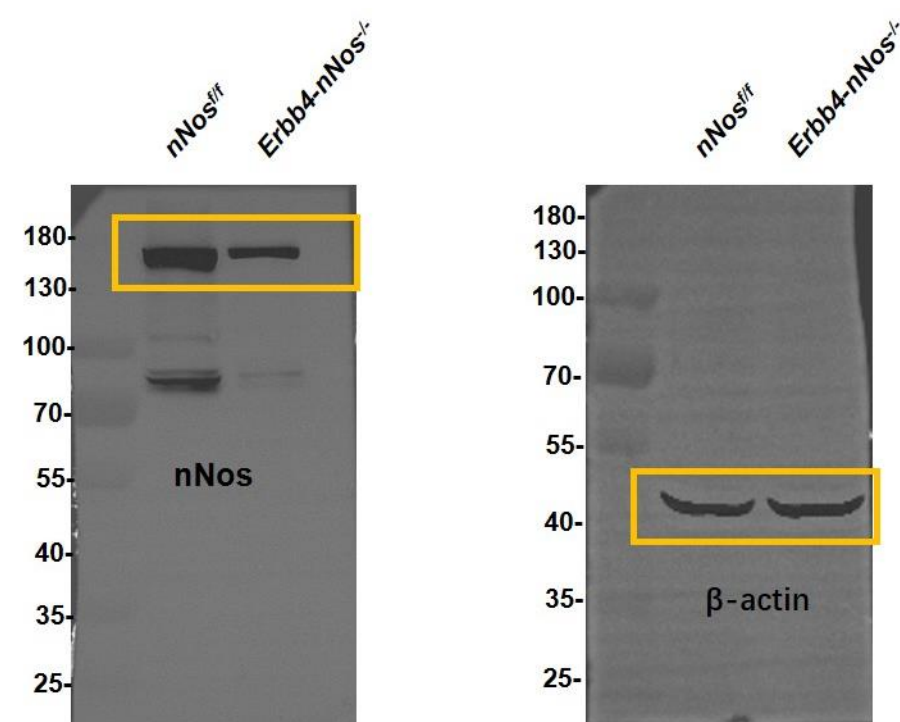

Supplement: Supplementary file 3 — Supplemental Material-western blots [file 41419_2024_6557_MOESM3_ESM.pdf]
